# Adaptation in auditory processing

**DOI:** 10.1152/physrev.00011.2022

**Published:** 2022-09-01

**Authors:** Ben D. B. Willmore, Andrew J. King

**Affiliations:** Department of Physiology, Anatomy and Genetics, University of Oxford, Oxford, United Kingdom

**Keywords:** adaptation, adaptive coding, auditory, cortex, sound statistics

## Abstract

Adaptation is an essential feature of auditory neurons, which reduces their responses to unchanging and recurring sounds and allows their response properties to be matched to the constantly changing statistics of sounds that reach the ears. As a consequence, processing in the auditory system highlights novel or unpredictable sounds and produces an efficient representation of the vast range of sounds that animals can perceive by continually adjusting the sensitivity and, to a lesser extent, the tuning properties of neurons to the most commonly encountered stimulus values. Together with attentional modulation, adaptation to sound statistics also helps to generate neural representations of sound that are tolerant to background noise and therefore plays a vital role in auditory scene analysis. In this review, we consider the diverse forms of adaptation that are found in the auditory system in terms of the processing levels at which they arise, the underlying neural mechanisms, and their impact on neural coding and perception. We also ask what the dynamics of adaptation, which can occur over multiple timescales, reveal about the statistical properties of the environment. Finally, we examine how adaptation to sound statistics is influenced by learning and experience and changes as a result of aging and hearing loss.

## Graphical abstract

**Figure:**
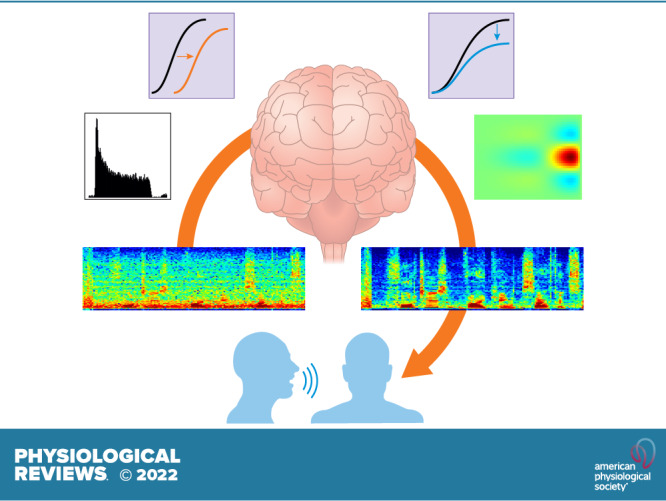

CLINICAL HIGHLIGHTS Adaptation is a fundamental process in the auditory system that dynamically adjusts the responses of neurons to unchanging and recurring sounds. This enables neuronal response properties to be matched to the constantly changing statistics of sounds that reach the ears.

Adaptive processes are critical to the successful outcome of auditory rehabilitation strategies: they enable users of hearing aids and cochlear implants to become accustomed to these devices. This has led to the development of targeted training regimes that harness the capacity of the brain to learn to use the more limited information provided by these neuroprosthetic devices. Adaptive processes also facilitate recovery from other forms of central nervous system injury and disease.

Adaptation triggered by hearing loss can also be maladaptive, giving rise to conditions such as tinnitus or hyperacusis.

Adaptation in the auditory system has been reported to be altered in dyslexia, while abnormalities in the mismatch negativity, the evoked response elicited by the brain in response to a deviant event occurring amidst a stream of repeated or familiar events, suggest that adaptive processing may also differ in schizophrenia and other neurodevelopmental disorders.

In this review, we discuss the regions of the brain where auditory adaptation occurs, the neuronal mechanisms underlying adaptation, and the impacts of adaptive processes on the neural coding and the perception of sound.

We also examine how adaptation to sound statistics is influenced by learning and experience and how it changes as a result of aging and hearing loss.

These findings illustrate how auditory processing in the brain constantly adjusts to enable neurons to operate across a wide range of environmental conditions and to accommodate intrinsic changes in inputs, including those arising from developmental changes in body anatomy and from impairments in sense organ function.

## 1. INTRODUCTION: DEFINING ADAPTATION

Adaptation is a very widely used term in neuroscience, reflecting the ubiquity of this process and its fundamental importance both for the way organisms interact with their environments and the operation of individual neurons. In the context of perception, a primary goal of sensory processing in the brain is to provide a neural basis for making accurate and reliable judgments and behavioral responses. Because our sensory environments are constantly changing, however, it is essential that the brain is able to analyze these signals in a flexible and adaptable fashion. A particular challenge is posed when the statistics of natural environments change, which can take place very quickly and over very wide ranges. This happens, for example, when we step out from a crowded, noisy bar onto a quiet street or enter a dark room from a bright, sunlit space. In doing so, we can experience changes in loudness or brightness of several orders of magnitude as well as changes in other statistical properties that reflect the particular characteristics of these environments. To deal with these variations in inputs, which may exceed the range of stimulus values that can be encoded by variations in their firing rate, sensory neurons exhibit compensatory changes in their sensitivity or tuning properties (1, 2). As a result of this so-called adaptive coding, these neurons are able to transmit information to their downstream targets more effectively, facilitating our ability to understand speech or recognize a face across a wide range of environmental conditions.

Adaptive processes also enable the brain to accommodate longer term changes in sensory inputs, particularly during development when the growth of the body can gradually alter the sensory information available at different ages. An extreme example is provided in the frog *Xenopus laevis*, where the laterally facing eyes of the tadpole move forward on the head during metamorphosis to establish a substantial region of binocular overlap. Vision plays a guiding role in bringing neural maps of space from the two eyes into register in the *Xenopus* optic tectum (3, 4) and in mammals is required for matching the orientation preference through each eye of binocular neurons in the developing visual cortex (5).

Another well-studied example of experience-dependent adaptive plasticity is found in the neural circuits responsible for sound localization. The physical separation of the ears gives rise to interaural differences in sound level and arrival time, while monaural spectral cues are generated when sounds enter the external ear, the visible part of the ear on the side of the head. These cues vary with the direction of the sound source relative to the head and that relationship changes during development as the head grows (6–11). It can also be altered by plugging one ear in young animals, which results in compensatory changes in auditory spatial processing in the brain and largely normal localization accuracy (12–17). Similarly, spatially conflicting visual information can induce corresponding shifts in auditory spatial tuning in the barn owl optic tectum (18) and its homolog, the mammalian superior colliculus (19). This cross-modal plasticity is beneficially adaptive in so far as it results in the emergence during development of topographically aligned maps of auditory and visual space (18, 19), although it can lead to systematic errors in auditory localization (20).

These findings highlight the functional value of experience-dependent plasticity during early life: it enables the developing brain to be calibrated so that best use is made of sensory inputs that vary with individual differences in the anatomy of the body. The capacity to adapt to altered sensory cues is not restricted to the developing brain, however, but continues into adulthood as well. Again, this has been demonstrated most clearly in the context of spatial hearing, where adult individuals can rapidly learn to localize accurately when they experience abnormal binaural cues (21–26).

On a shorter timescale, the tuning properties of auditory neurons undergo changes according to the behavioral context in which sounds are presented, emphasizing particular stimulus features in a task-relevant fashion (27–30). This indicates that auditory processing is continually adjusted during active listening in ways that optimize^1^ the processing of those features to which attention is drawn, thereby facilitating sound detection and discrimination (31). Adaptive changes in neuronal stimulus selectivity also occur during the acquisition and reversal of conditioned behavioral responses (32–34) and have often been associated with the training-induced improvements in discrimination that underlie perceptual learning (35–37).

From a clinical perspective, adaptive processes in the brain appear to be critical to the successful outcome of auditory rehabilitation strategies by enabling users of hearing aids and cochlear implants to become accustomed to the novel inputs provided by these devices (38–42), as well in the recovery from other forms of central nervous system injury and disease (39). That said, the neural plasticity triggered by hearing loss can sometimes be considered to be maladaptive, giving rise to conditions such as tinnitus or hyperacusis (42, 43).

Together, these findings illustrate how auditory processing in the brain constantly adjusts to enable neurons to operate in a task-specific fashion across a wide range of environmental conditions and to accommodate intrinsic changes in inputs, including those arising from developmental changes in body anatomy and from impairments in sense organ function. While adaptive processes take place over multiple timescales, the primary focus of this article is on the rapid, stimulus-driven adaptation that is critical for perception in different soundscapes. We start by considering adaptation as a change in neuronal response over the course of a constant sound. Although such adaptation is clearly important for many forms of sensation, such as being oblivious to the persistent stimulation provided by our clothing, constant sounds are relatively rare in nature, so this form of adaptation is an edge case. As the sophistication of experimental investigation of auditory adaptation has increased, it has become clear that neurons adapt not only to the presence of a constant stimulus but also to the statistical properties of changing stimuli: the mean sound level, sound contrast, and higher order statistics that characterize real soundscapes. We examine how these forms of adaptation affect neuronal response properties, as well as their role in enabling neurons to represent sounds efficiently (44) and, in combination with attentional modulation, in representing sounds in noisy environments (45), thereby contributing to auditory scene analysis (46). We next consider how adaptation affects neuronal responses to recurring sounds. Presentation of “deviant” sounds within a stimulus sequence has revealed the stimulus-specific nature of this form of adaptation, with research in this area providing insights into the neural encoding of temporal regularity in sound sequences and the role of prediction in sensory processing.

As in other sensory systems, neuronal responses are affected by adaptation throughout the auditory system. We examine whether different forms of adaptation are implemented locally or inherited by neurons from their upstream sources of input and the extent to which these properties are organized hierarchically along the auditory pathway and influenced by descending projections that transmit signals back to earlier processing levels. Finally, we consider how these adaptive processes change over the life span, how they are affected by experience and hearing loss, and how abnormalities in adaptation might contribute to neurodevelopmental conditions, such as dyslexia. While our focus throughout is on adaptation in the mammalian auditory system and how this impacts human perception, we also include some studies in avian species, which suggest that, as with other aspects of hearing (47), comparable adaptive mechanisms may have evolved independently in each case.

## 2. FIRING-RATE ADAPTATION

Firing-rate adaptation is a fundamental property of sensory neurons and is defined by a reduction over time in the number of impulses elicited by a constant stimulus. Reports of adaptation date back to recordings of the responses of cat plantar digital nerves to tactile stimulation of the toe (48). Because individual sensory neurons typically encode a limited range of stimulus intensities, their dynamic range, by a change in firing rate, adaptation helps to prevent their responses from being saturated when a new stimulus is encountered. The existence of adaptation indicates that neurons are more sensitive to novel or transient stimuli than to constant background stimulation. At a perceptual level, adaptation similarly results in a loss of sensitivity to a persistent stimulus, which, particularly in the case of vision, can be associated with a temporary aftereffect when that stimulus is removed (49).

### 2.1. Firing-Rate Adaptation in the Auditory Nerve

In the auditory system, firing-rate adaptation is first exhibited at the level of the auditory nerve fibers, which transmit information about the auditory environment from the cochlea in the inner ear to the auditory brainstem. Within the cochlea, the different frequency components of a sound are separated by the mechanical tuning properties of the basilar membrane, so that higher frequency tones cause maximum vibration at the base of the cochlea and lower frequencies more toward its apex. In mammals, the organ of Corti rests on the basilar membrane and contains two types of mechanosensory receptor cells, inner and outer hair cells, which are arranged in rows along the cochlea. The great majority of the auditory nerve fibers each innervate a single inner hair cell and show equivalent frequency tuning, reflecting their point of innervation along the cochlea.

**FIGURE 1.**
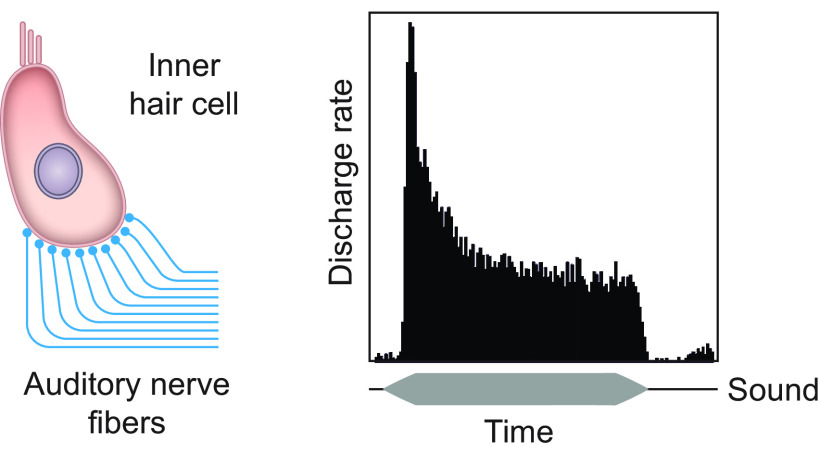
Firing-rate adaptation in the auditory nerve. The majority of auditory nerve fibers innervate individual inner hair cells in the cochlea, with ∼10–20 afferent neurons forming excitatory synaptic connections with each inner hair cell (*left*). The peristimulus time histogram on the *right* illustrates the pooled response of an auditory nerve fiber to multiple presentations of a ramped pure tone stimulus (depicted by the horizontal gray bar below the plot). The instantaneous firing rate is highest when the sound is first presented, after which it adapts very rapidly and then more slowly to a steady-state firing rate. Following stimulus offset, the firing rate typically declines below the spontaneous baseline level and then recovers from adaptation.

Electrophysiological recordings in a range of species have shown that presentation of a constant tone that falls within an auditory nerve fiber’s frequency response area produces an increase in action potential firing rate that reaches a maximum soon after stimulus onset and then declines rapidly over the first few milliseconds, followed by more gradual adaptation toward a steady-state level (50–55) (FIGURE 1). The offset of the sound is then associated with a temporary drop in spike frequency below the spontaneous or baseline rate of firing. This pattern of spiking activity appears to be well suited for representing the rapid variations in sound level that are characteristic of many natural sounds and for filtering out redundant information. Interestingly, in the chick auditory nerve, rate coding becomes noisier, and therefore less reliable, as the response declines during adaptation, whereas the precision of spike timing remains unchanged, highlighting the likely importance of high temporal fidelity in the signals transmitted to the brain by afferent auditory fibers (56).

### 2.2. Mechanisms of Adaptation

In general, firing-rate adaptation can arise from a neuron’s intrinsic ion channels or from a change in its synaptic inputs or can be imposed by the adaptive properties of the upstream neurons (for a more detailed review, see Ref. 1). As in other sensory systems, adaptation occurs as part of the transduction process carried out by the hair cells in both the auditory and vestibular organs of the inner ear (57). Displacement by an external force (e.g., sound or head motion/tilt) of the stereocilia or hair bundles that give these receptor cells their name alters the tension in the tip links that connect adjacent rows of stereocilia. This modulates the opening of mechanoelectrical transduction channels, producing graded changes in membrane potential as a result of the influx of predominantly potassium ions.

Studies in several species have shown that a sustained deflection of the hair bundle in the direction of the tallest stereocilia causes the mechanoelectrical transduction channels to open and then close again, resulting in a decline in the initial inward current. While fast and slow mechanisms for this form of adaptation have been identified in low-frequency hair cells, including those in the vestibular parts of the inner ear or in the basilar papilla, the nonmammalian hearing organ, transducer adaptation in mammalian auditory hair cells seems to operate in a different way (57, 58). Indeed, the sound-evoked receptor potentials recorded from mammalian inner hair cells appear to be too sustained to account for adaptation in the auditory nerve (59, 60). Instead, synaptic depression occurs at the ribbon synapses between the hair cells and the afferent nerve fibers, indicating that adaptation is due primarily to synaptic fatigue (61–63). Compared to those measured for the auditory nerve, some differences in the time constants of firing-rate adaptation have been reported at higher levels of the auditory pathway, which presumably reflect the additional contribution of intrinsic and network properties (64–66).

### 2.3. Firing-Rate Adaptation in the Presence of Background Noise

Another important consequence of firing-rate adaptation is that this can affect the responses of neurons to sequential sounds (which we discuss in more detail in sect. 4). For example, prior exposure to a brief tone or noise burst reduces the response of auditory nerve fibers to a subsequent tone (67, 68). More intriguingly, the presence of continuous noise beginning before the onset of a tone reduces the responses of auditory nerve fibers to that stimulus and shifts their dynamic ranges, enabling them to encode sounds at levels that would saturate their responses in quiet conditions (69). Dynamic range adaptation in the presence of continuous background noise is also observed in the central auditory pathway (70–72) and is likely to be related to the improvement in speech-in-noise recognition reported if the masking noise starts before the speech sounds rather than at the same time (73, 74).

### 2.4. Antimasking and the Medial Olivocochlear Reflex

Because behaviorally relevant sounds usually occur against a background of other sounds in natural listening conditions, it is important to understand the basis for these findings. One factor that is often thought to contribute to enhanced speech perception following adaptation to background noise is the medial olivocochlear reflex, which allows central control over sound processing in the auditory periphery (75). Medial olivocochlear neurons are located in the superior olivary complex in the brainstem and have efferent projections that mainly innervate outer hair cells in the contralateral cochlea. Release of acetylcholine from medial olivocochlear neurons inhibits the electromotility exhibited by outer hair cells, which is responsible for the mechanical amplification of low-level sounds (76). Recordings of auditory nerve responses with and without crossed olivocochlear bundle stimulation suggest that these descending signals improve the discriminability of sounds in the presence of broadband noise by restoring the dynamic range of auditory nerve fibers in noise to the values observed in quiet (77, 78).

Whether olivocochlear efferents have an antimasking role has been questioned, however, at least in humans, by more recent evidence demonstrating that noise adaptation can be reduced by introducing statistical fluctuations into the noise, even though the medial olivocochlear reflex was unaffected (79). Furthermore, measurements of otoacoustic emissions, the sounds generated as a by-product of cochlear amplification, suggest that postadaptation improvements in sensitivity to amplitude modulation for tones presented in noise are unlikely to result from an efferent-dependent reduction in cochlear responses (80). Finally, noise adaptation during word recognition is exhibited by cochlear implant users, who are not thought to possess a functioning medial olivocochlear reflex (81). Thus, although peripheral mechanisms clearly contribute to adaptive coding in the auditory system, whether descending modulation of cochlear gain by medial olivocochlear neurons plays a critical role in speech-in-noise perception remains controversial.

While these studies have demonstrated that dynamic range adaptation takes place in the presence of continuous background noise, adaptation to statistical variations in the sounds reaching the ears also plays an important role in this process, as will be discussed in sect. 3.

## 3. ADAPTATION TO SOUND STATISTICS

Sensory systems have to contend with real-world stimuli whose statistics are constantly varying, and in multiple sensory systems, initially vision (82), researchers have investigated neuronal adaptation to these changing statistics. This work has revealed diverse forms of adaptation to stimulus statistics, which are now viewed as fundamental to the way that sensory information is encoded in the brain. Such adaptation has theoretical implications; for example, it may enable the brain to represent sensory information in ways that are computationally efficient and robust to noise. In the auditory system, certain forms of adaptation to stimulus statistics have been well studied and are known to be present at early sensory processing levels. For example, adaptation to mean sound level has been demonstrated at levels from the auditory nerve upward. Adaptation to more complex statistics is also present at higher levels. Together, these forms of adaptation subserve a variety of functions in sound perception and may enable efficient neuronal representation of sounds.

### 3.1. Adaptation to Mean Sound Level

A fundamental challenge for the auditory system is how to encode sound levels over a range of more than 120 dB (83). Decibels are a logarithmic measure, such that a 10-fold multiplicative increase in sound pressure (measured in Pascals) produces an additive increase of 20 dB; thus a 120-dB range corresponds to a sound pressure range of 1,000,000. Over much of this range, the human auditory system can discriminate sound-level differences of ∼1 dB (84). Because of the refractory period of action potentials, individual neurons can modulate their firing rates only over a much smaller range of values (up to 500 Hz) and even neuronal population codes have much smaller dynamic ranges, estimated at 30 to 40 dB in the auditory nerve (84, 85), compared to that found perceptually. A coding strategy is therefore required if the entire 120-dB range is to be represented with sufficient accuracy to support human discrimination abilities.

One strategy, known as range fractionation, would be for different parts of the dynamic range to be represented by distinct subpopulations of neurons. Indeed, such a strategy is employed in the auditory nerve, where three classes of fibers have been described that appear to encode the same sound frequencies but over different portions of the range of possible sound levels (86, 87). These fibers vary in their spontaneous firing rates, with the high spontaneous firing-rate auditory nerve fibers having the lowest thresholds and saturating rate-level functions, whereas the medium and low spontaneous rate fibers have higher thresholds and wider dynamic ranges. However, this strategy implies that, at any given sound level, only a subpopulation of neurons will provide meaningful coding, others will be either below or above their dynamic ranges. Also, at high sound levels, most of the auditory nerve fibers will produce large numbers of action potentials (and thereby consume energy), but, due to saturation, those with lower thresholds will convey relatively little information in their firing rates.

**FIGURE 2.**
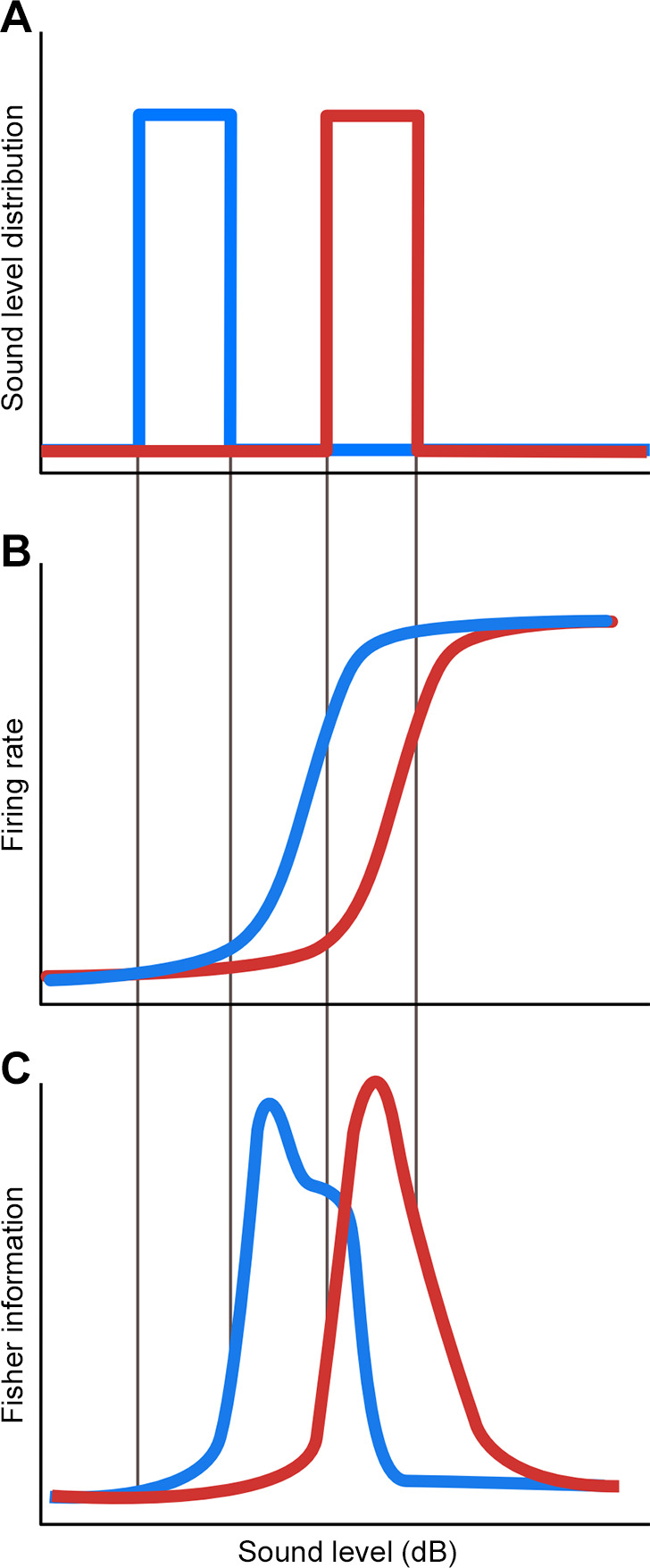
Adaptation to mean sound level in guinea pig inferior colliculus neurons. *A*: sounds with different mean levels (blue, low; red, high) were presented to guinea pigs. *B*: shift in the dynamic range of neurons in response to sounds with the sound-level distributions shown in *A*. Neuronal responses to the low-level sound tended to have lower thresholds than neuronal responses to the high-level sound. *C*: when the responses of the population of neurons were analyzed, these shifts in threshold were found to enhance the Fisher information for the sound levels presented in each condition, indicating that adaptation acts to increase the information encoded by neurons about the level of recently heard sounds. Modified from Ref. 88, with permission from *Nature Neuroscience*.

Adaptation provides a potential solution to this problem by shifting the dynamic range of neurons to enable a larger proportion of the neuronal population to participate in coding sounds of all levels. Such adaptation was first demonstrated in the midbrain of guinea pigs (88, 89) and was later found in the auditory nerve too (90, 91). The level of natural sounds is correlated over time (for example, due to being in a noisy environment), so that, at least for short time periods, sound levels tend to vary over a limited range. Electrophysiological recordings in guinea pigs have demonstrated that neurons in the inferior colliculus (IC), the principal auditory midbrain nucleus, adjust their responses to the mean (as well as the variance and more complex statistics) of sound-level distributions, moving the dynamic range of individual neurons towards the range of recently heard sound levels and improving the accuracy of the neuronal population code for those levels (FIGURE 2).

These effects were found to be particularly salient in response to changes in mean sound level (88). In an idealized situation, when the mean sound level is high, neurons would shift their dynamic ranges upwards, to avoid being saturated by louder sounds. When the mean sound level is low, neurons shift their dynamic ranges downward, so they are more sensitive to quieter sounds. Because sound levels are correlated over time in natural sounds, this should allow much of the neuronal population to participate effectively in representing all sound levels, unlike the range fractionation strategy described above. Dean and colleagues (88) found that the true picture was more complex than this: changing the mean sound level affected different neurons differently, and IC neurons did not shift their thresholds below a baseline value. However, across the population, adaptation to the mean sound level behaved as expected, increasing coding accuracy (as measured by Fisher information) for the most frequently occurring sound levels.

In addition to dynamic-range adaptation to mean sound level, neurons show adaptation of their spectral filtering characteristics to sounds of different mean levels. This has been investigated in songbirds by recording neuronal responses in the primary auditory forebrain region field L to amplitude-modulated noise stimuli that capture the temporal frequency and amplitude distributions of natural sounds (92). This revealed that varying the mean level of the stimuli induced systematic changes in the shape of neuronal spectrotemporal receptive fields (STRFs). High-mean stimuli typically produced STRFs with narrower bandwidths and larger negative components. Low-mean stimuli produced STRFs with wider bandwidths and smaller negative components. These results suggest that, when the overall sound level is high, neurons adapt their responses to respond to local level differences, whereas when the sound level is low, they integrate sound over time.

#### 3.1.1. Mechanisms of dynamic-range adaptation to mean sound level.

An analogue of midbrain adaptation to sound level has been found in the auditory nerve of cats (90), though adaptive coding of sound level is weaker here than in the IC. This suggests that adaptation in the midbrain is not merely inherited from the auditory nerve and is instead organized hierarchically, with neurons higher in the auditory system adapting more completely than those lower down.

The presence of dynamic range adaptation in the auditory nerve raises the question of the relationship between the mechanisms underlying adaptation to mean sound level and classical firing-rate adaptation. Firing-rate adaptation would be expected to occur in experimental paradigms that investigate adaptation to mean sound level, and it is possible that the two types of adaptation are subserved by related mechanisms. Although some similarities certainly exist, only a weak correlation was found in the auditory nerve between the decline in firing rate and the size of dynamic range shifts to different sound-level distributions, hinting that these processes may be distinct from one another (90). However, further work elicited both firing-rate and dynamic-range adaptation to mean sound level in auditory nerve fibers and fitted a dual-adaptation phenomenological model to these responses (91). This showed that the time constants of the two adaptive processes, i.e., how rapidly neurons adapt after a change in stimulus, were similar (100–400 ms) and that these time constants were correlated across auditory nerve fibers, suggesting a common mechanism for these two forms of adaptation.

Another possibility (raised by Ref. 89) is that dynamic-range adaptation observed at higher levels of the auditory system may be the result of firing-rate adaptation at lower levels of the pathway. Again, this issue has been probed by measuring the time constants of adaptation. Midbrain neurons show both rapid adaptation to mean sound level with time constants of around 160 ms and also much slower adaptation on the timescale of tens of seconds (89). The faster adaptation is compatible with an explanation in terms of synaptic depression (93) or medial olivocochlear efferent effects (76, 94), which can also operate over hundreds of milliseconds. Although the slower adaptation fits less well with observed synaptic depression, it is still compatible with medial olivocochlear effects, which can also operate over long timescales. As discussed above, however, recent work has cast doubt on the role of the medial olivocochlear reflex in mediating noise adaptation when listening to speech in noise, whilst supporting the idea that this relies on dynamic-range adaptation to the most frequently experienced sound levels (79).

Investigation of the time course of adaptation to mean sound level in the guinea pig IC in response to a stimulus that switched repeatedly between high and low mean levels (95) has shown that the expected adaptation to mean level occurred more rapidly as exposure to the switching stimulus increased (“meta-adaptation”). It therefore appears that auditory midbrain neurons can adjust their response properties to the statistics of auditory environments more rapidly if those environments have been repeatedly experienced before. This study also found that meta-adaptation in the IC was attenuated by cooling of the cortex during the recordings, suggesting that the ability to rapidly respond to statistical changes in the environment has a top-down, cortical origin.

### 3.2. Adaptation to Sound Contrast

**FIGURE 3.**
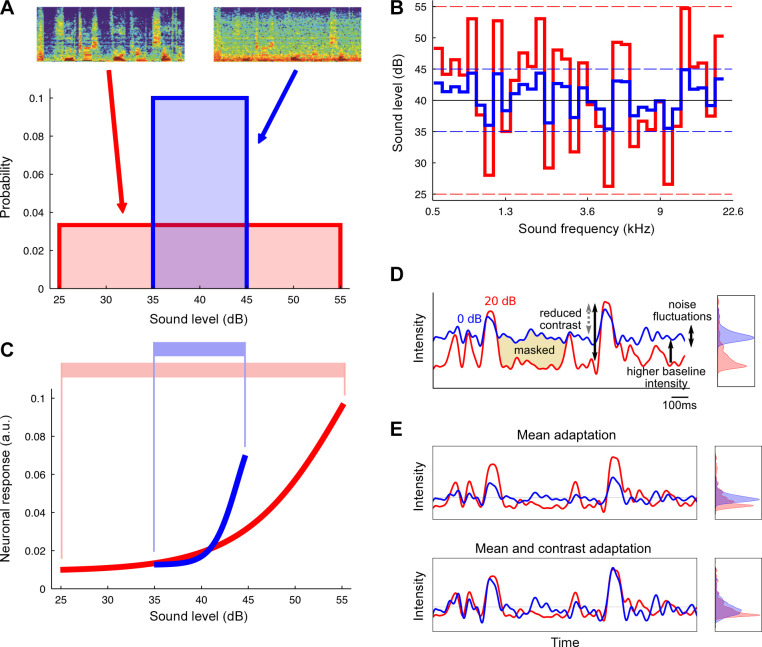
Contrast gain control in ferret auditory cortical neurons. *A*: sounds of high (30-dB range, red) and low (10-dB range, blue) contrasts were used, which are analogous to speech presented in isolation (high contrast) and against a background of noise (low contrast); spectrograms shown in upper inset panels. *B*: slices taken through the spectrograms of these sounds at a single moment in time show that the sounds had identical spectrotemporal structure except for their contrasts. *C*: neuronal responses were measured during the presentation of each of the sounds. The aspect of the responses that varied most strongly and consistently was the gain, the slope of the response-level curves, illustrated schematically here. For the low contrast sound (blue), neuronal gain was high, indicating that the neuron was more sensitive to a change in sound level under this condition. For the high contrast sound (red), neuronal gain was low, indicating that sensitivity had decreased. These changes broadly matched the sound-level range of the sounds (shaded areas). *D*: in subsequent experiments, human speech was presented either on its own (“clean,” red) or against a background noise (“noisy,” blue); spectrograms in *A*. The effect of the noise is to decrease the contrast of the overall sound, and to mask parts of the speech. This results in distributions of sound levels that are quite different between the 2 conditions (*right inset*). *E*: the effect of adaptation to mean sound level (*top*) and combined adaptation to mean and contrast (*bottom*) is to reduce the effect of the noise on neuronal responses, resulting in distributions (*right insets*) that are more similar between clean and noisy conditions. In this way, neuronal adaptation results in a degree of invariance to background noise. Modified from Refs. 96, 97 (in accordance with http://creativecommons.org/licenses/by/4.0/).

The variance of sound level is another important statistic that can change rapidly over time. For example, a naturally varying sound (such as a human voice) with a large dynamic range will have high sound-level variance when heard in silence. However, when heard against a constant noise background, the same sound will have lower variance (96) (FIGURE 3, *A*–*C*). Such changes in variance might be expected to induce neuronal adaptation. This has been investigated using amplitude-modulated pure-tone stimuli (98), showing that neurons in cat IC adapt their responses according to the depth of modulation. Measurement of adaptation to sound-level variance of complex stimuli in the guinea pig IC showed only modest effects in individual neurons (88). However, across the neuronal population, coding accuracy improved as a result of variance adaptation, similar to the effects observed (in the same study) for adaptation to mean sound level.

Neurons in field L of songbirds do not adapt their spectral filtering properties in response to changes in the variance of sound level (unlike changes in mean sound level) (92). However, increasing the sound-level variance generally decreased the gain of neuronal responses. This suggests that neurons may adjust their gain (sensitivity to changes in sound level) in a way that compensates for changes in variance, so that neurons tend to respond more strongly to changes in sound level when presented in a low-variance context and less strongly to the same change in a high-variance context. Additionally, such adaptation was found to alter the range of stimulus levels over which neurons can detect fluctuations in the stimulus, analogous to the changes previously observed in guinea pig IC (88).

A related paradigm has been used to investigate adaptation to sound contrast in the primary auditory cortex (A1) of ferrets (97) (FIGURE 3). The role of contrast in visual coding has been extensively investigated, and adaptation to visual contrast is known to be widespread in the visual system (99). In vision, contrast can be defined as the variance (or change) in luminance across a stimulus, divided by the mean luminance of the stimulus. In the auditory system, contrast could similarly be defined as the variance of a sound divided by its mean. However, since perceptual and neuronal responses to sounds are typically closer to logarithmic functions of sound pressure than linear ones, it is more natural to define auditory contrast as the variance of sound level, measured on a logarithmic scale (in dB). These two definitions are closely mathematically related (97).

Rabinowitz et al. (97) investigated the responses of neurons in ferret A1 to dynamic random chords whose contrast was systematically varied (FIGURE 3). They found strong, consistent adaptation of the slope of the function depicting the relationship between firing rate and sound level for these neurons. This adaptation can be conceptualized as changes in response gain, such that neuronal gain is higher in low-contrast conditions and lower in high-contrast conditions. Across the neuronal population, these changes in gain were highly systematic, confirming that contrast gain control takes place in the auditory cortex. To investigate the circuit basis for adaptive coding, this work was subsequently extended to the mouse auditory cortex, where neurons exhibit similar contrast-dependent gain changes (100). Optogenetic investigation of the cortical circuitry has shown that parvalbumin-positive interneurons, which have been shown to control the activity of excitatory pyramidal neurons in visual cortex (101), are similarly involved in regulating auditory cortical gain (102, 103), but are not responsible for contrast-dependent gain changes (102). However, recent evidence indicates that contrast gain control in mouse A1 is dependent on synaptic zinc signaling (104).

There are two sources of variability in spectrotemporal sound stimuli. First, in any given frequency band, the level of natural sounds may vary over time (temporal contrast). Second, at any moment in time, the sound level of different frequencies may vary (spectral contrast). Systematic changes in both of these properties may produce corresponding changes in sound-level variance, which might be expected to induce adaptation. Using stimuli that varied independently in their temporal and spectral contrast, contrast gain control in the auditory cortex was found to depend largely on contrast within the receptive field of the neuron under investigation (105), suggesting that the spectral range over which each neuron integrates contrast is similar to that over which sound level influences that neuron.

#### 3.2.1. Contrast adaptation across the auditory pathway.

**FIGURE 4.**
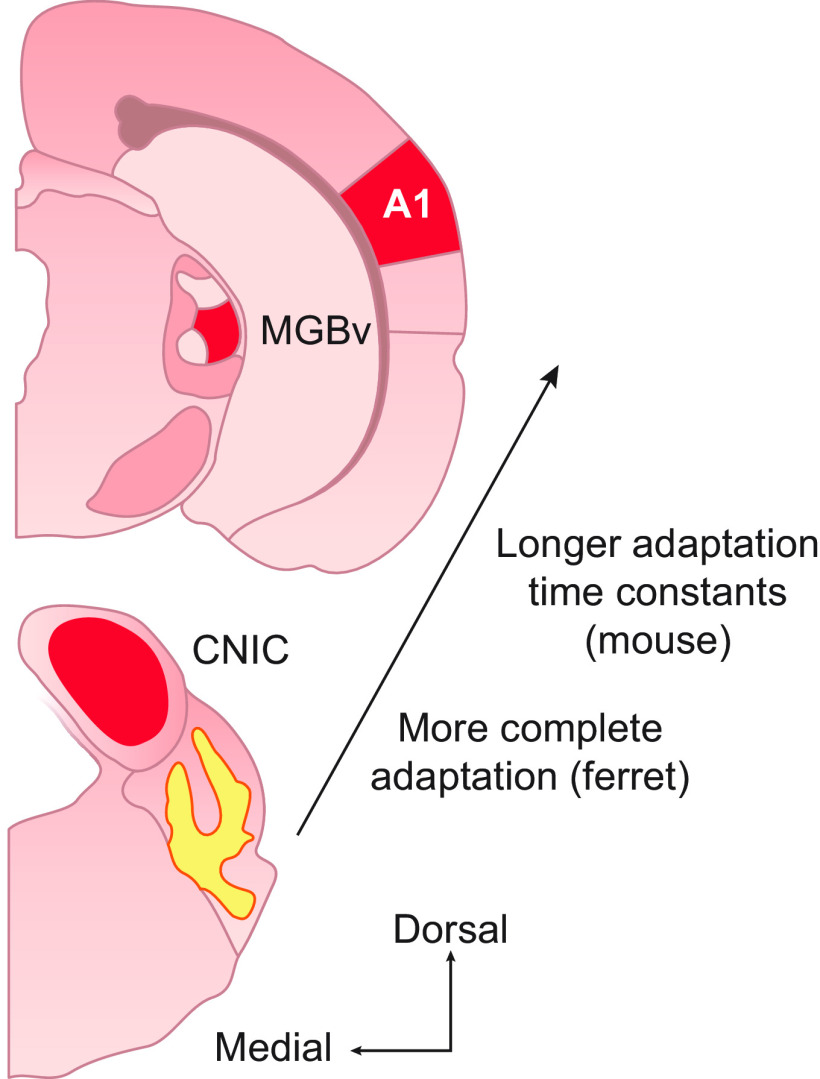
Distribution of contrast gain control in the auditory system. Two coronal sections of the mouse brain are represented, with lemniscal areas of the midbrain, thalamus and cortex where contrast gain control has been found colored in red. Contrast adaptation is found to some degree in the central nucleus of the inferior colliculus (CNIC) of both mice and ferrets. In mice, adaptation is strong at this level of the auditory hierarchy, and largely compensates for changes in stimulus contrast. At higher levels of the mouse auditory system [the ventral division of the medial geniculate body (MGBv) and primary auditory cortex (A1)], the time constants of adaptation are slower. Subcortical adaptation is independent of descending cortical input. In ferrets, contrast adaptation increases in strength and consistency from CNIC to A1. In both cases, these findings are indicative of a hierarchy of contrast adaptation in the auditory pathway.

The robust nature of contrast-dependent adaptation in the auditory cortex raises the question of whether similar adaptation is present in, or even inherited from, subcortical regions. This is an important question since adaptation to mean sound level is found in both the auditory nerve and the IC. Recordings from ferret IC have shown that contrast adaptation is less robust in the midbrain than in cortex, and modeling studies suggest that it is present only to a limited degree in the auditory nerve (96). A slightly different picture has emerged in mice, however, where neurons in both the auditory midbrain and thalamus show consistent, compensatory gain control, more similar to the auditory cortex of both species (106) (FIGURE 4). These recordings were made in the central nucleus of the IC (CNIC) and the ventral division of the medial geniculate body (MGBv), indicating that contrast gain control is a feature of neurons in the tonotopically organized core or lemniscal projection to A1.

In both ferrets and mice, the time constants of adaptation to sound contrast tend to increase along the auditory pathway (96, 106). This suggests that contrast adaptation at higher levels of the pathway is not simply inherited from neurons at lower levels in either species. Furthermore, experiments involving optogenetic suppression of cortical activity suggest that contrast adaptation is not simply passed back down the hierarchy from cortex to subcortical areas: the degree of adaptation to sound contrast in IC and MGB is unchanged in the absence of cortical activity (106). This is consistent with a lack of effect of cortical cooling on adaptation to mean level by IC neurons in guinea pigs (95). As mentioned in sect. 3.1.1, however, cortical inactivation does slow down sound-level adaptation by IC neurons when the same sound statistics are reencountered (95), raising the intriguing possibility that descending projections may also contribute to adaptation to other stimulus statistics, including contrast, in rapidly changing acoustic environments.

These findings suggest that relatively independent mechanisms of adaptation to stimulus contrast operate at multiple levels of the auditory system. This is consistent with a hierarchy of adaptation to which each area contributes so that higher areas adapt more completely than lower areas, as is clearly the case in ferrets. The lengthening time constants may also reflect this computation becoming more complete at higher stages of the hierarchy. Alternatively, it is possible that different time constants are appropriate for the distinct functional roles of regions at different levels of the hierarchy.

### 3.3. Adaptation to More Complex Aspects of Sound

In addition to the mean and variance of sound level, adaptation to higher order properties of sound has also been studied. This includes both statistically defined properties as well as properties inspired by perceptual properties of sound. Sensitivity to higher order sound-level statistics has been probed by measuring adaptation of neurons in guinea pig IC to stimuli with bimodal sound-level distributions (88). Although individual neurons did not produce bimodal adaptation, the neuronal population response encoded the high-probability regions with the highest accuracy. Similarly, cat IC neurons adapt to the kurtosis of the modulation envelope of sounds, even when the mean and variance are held constant (98). Although based on a small number of studies, these results raise the possibility that neurons may be sensitive to a wide range of higher order sound statistics.

Perceptual experiments have demonstrated adaptation to both simple and complex aspects of sound. Experiments involving adaptation to timbre in human listeners have used morphed continua of sounds (107). To induce adaptation, a sound from one end of a continuum was presented repeatedly. When adaptation was subsequently probed using a neutral morph in the middle of the continuum, the percept of the neutral morph was shifted away from the repeatedly presented sound. Another perceptual phenomenon that may be related to neuronal adaptation is forward masking, where the threshold of a sound is raised by a preceding masker. Psychophysical evidence for forward masking for amplitude-modulated signals, which are an important component of speech and other natural sounds, is generally not replicated in the responses of neurons in the rabbit IC (108). However, in the squirrel monkey auditory cortex, presentation of amplitude-modulated tones suppressed responses to subsequent amplitude-modulated tones, and this effect was coarsely tuned for the modulation frequency (109). Although consistent with a hierarchy of adaptation for this feature across the auditory system, it is possible that forward masking may instead be due to temporal integration producing more persistent responses to the masker at higher processing levels (110), which is supported by recordings from the auditory cortex (111).

### 3.4. Adaptation to Spatial Statistics

A very important function served by several of our senses, including audition, is to localize objects in our surroundings. As we described in sect. 1, the neural circuits responsible for determining sound-source direction are shaped by experience, allowing them to be calibrated according to the anatomy of the head and external ears and to adapt to hearing loss-induced changes in the spatial cues available. The existence of this adaptive plasticity indicates that the response properties of auditory neurons can be adjusted in both developing and adult animals in ways that preserve accurate sound localization. As with other aspects of auditory function, neuronal sensitivity to auditory spatial cues and the perception of sound-source location are also continually updated in a context-dependent way. In addition to providing another example of the dependence of auditory processing on the prevailing sound statistics, these findings have provided important insights into the principles that govern spatial hearing in mammals. In particular, studies of adaptation to the recent history of sound stimulation have challenged the view that maintaining a neural representation of absolute sound-source location is the primary function of auditory spatial processing in the brain.

**FIGURE 5.**
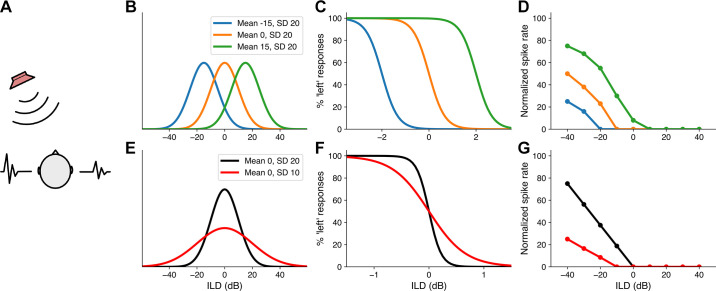
Adaptation to spatial statistics. *A*: sounds originating from locations to one side of the head reach the ears at slightly different times, resulting in interaural time differences (ITDs). At relatively high frequencies, they also generate interaural level differences (ILDs). This is illustrated by the differences between the sound waveforms reaching each ear. ITDs and ILDs are therefore the binaural cues used to localize sound sources in the horizontal plane. *B*: adaptation stimuli comprising broadband noise sequences in which the ILD rapidly fluctuated according to 1 of 3 Gaussian distributions with the same variance (SD = 20 dB) but with different means [−15 dB (higher in the left ear), 0 dB (equal in the 2 ears), or +15 dB (higher in the right ear)]. *C*: psychometric functions showing perceptual ILD sensitivity of a human listener for test stimuli presented over headphones following adaptation to each of these distributions. *D*: relationship between normalized firing rate and ILD for neurons recorded in the left IC of an anesthetized ferret for the same 3 ILD distributions. Note that the perceived laterality and neuronal response properties shift as the mean of the ILD distribution is changed. *E*: adaptation stimuli with the same mean (0 dB), but different variances (SD = 20 or 10 dB, changing the range of values presented). *F* and *G*: the human psychometric functions (*F*) and neuronal rate-ILD functions (*G*) both show improvements in spatial sensitivity when the variance decreases. Modified from Ref. 117 (in accordance with http://creativecommons.org/licenses/by/4.0/).

A number of psychophysical studies in humans have shown that presentation of a sound at one location can induce a shift in the perceived location of subsequent sounds (or in binaural cue sensitivity) away from that of the adapting stimulus (112–116). Similar findings have been obtained by presenting listeners with sequences of broadband noise over headphones in which the interaural level difference (ILD) fluctuated according to a Gaussian distribution, generating the percept of a lateralized sound that rapidly changed location (117). When the mean of the adapting ILD distribution was displaced to the left or right, the perceived location of a subsequent sound became biased in the opposite direction, whereas altering the variance of the distribution to either increase or decrease the range of possible locations resulted in a corresponding change in perceptual sensitivity (FIGURE 5*A*). This study also showed that the position or gain of the firing-rate-ILD functions of ferret IC neurons changed in the same way when the mean or variance of the adapting ILD distribution was altered (117) (FIGURE 5*B*). The sensitivity of IC neurons in guinea pigs to interaural time differences (ITDs), the other binaural cue for sound-source location, also adapts to the mean of the preceding ITD distribution, with comparable perceptual changes again being observed in human listeners (118).

Not surprisingly, binaural cue sensitivity in the auditory cortex depends on preceding sounds too, but the adaptive processing found in the auditory midbrain largely mirrors the context-dependent effects on spatial hearing seen behaviorally in humans. Adaptation to spatial statistics does not originate in the IC, however, since it has also been shown to be a property of neurons in the auditory brainstem nuclei where sensitivity to ITDs and ILDs first arises. Mechanistically, dynamic coding of ITDs and ILDs in the brainstem is thought to be mediated by the activity-dependent release of the inhibitory neurotransmitter GABA acting via presynaptic GABA_B_ receptors to modulate the strength of inputs to neurons in the medial superior olive (MSO) (115) and lateral superior olive (LSO) (119), respectively. In addition, more rapid alterations in the relative strength of the inputs from each ear, resulting from dynamic range adaptation to changes in sound level, appear to contribute to ILD adaptation in the LSO (120) and therefore most likely in downstream auditory brain areas like the IC as well (117).

As with the IC recordings, when the paradigms used to demonstrate adaptive coding of ITDs in the MSO (115) and of ILDs in the LSO (120) were adopted in human psychophysical experiments, similar changes in auditory spatial discrimination were observed. For example, it has been found that dynamic range adaptation improved ILD resolution across the averaged responses of a population of LSO neurons for the spatial cue values that were most commonly encountered and resulted in corresponding, ILD-specific discrimination improvements in humans (120). Furthermore, a conclusion of most of the studies on adaptation to spatial statistics is that processing in the auditory system emphasizes relative differences between sound-source locations, rather than maintaining an accurate representation of absolute location (115–117, 120–122). This strategy should facilitate the segregation of multiple sound sources, including foreground sounds of interest from background sounds (113), in the current acoustical environment.

Another aspect of spatial statistics that needs to be considered is reverberation. This a ubiquitous feature of natural sounds, whereby sound that arrives directly from its source is accompanied by multiple delayed versions due to reflections off the walls and other surfaces. The resulting superposition of sounds is complex, but interpreting sounds correctly in the presence of reverberation is a daily necessity for effective hearing. A key factor enabling the reliable localization of sound sources in everyday reverberant environments is thought to be the precedence effect, whereby the responses of human listeners are dominated by the spatial cues associated with the first-arriving direct sound, rather than those associated with the slightly later reflected sound waves (123). Although both peripheral and central mechanisms have been implicated in the precedence effect, the IC appears to be a key stage in its generation (124). Other evidence points to the importance of ITDs in the early rising phase of modulated sounds, which is likely to be important for the localization of speech in noisy or reverberant conditions (125). In the latter case, suppression of later-arriving spatial information may involve adaptation of monaural inputs before the MSO (126).

Aspects of the precedence effect have been shown to build up or break down depending on the recent history of stimulation (127–130), suggesting that the auditory system adapts to the acoustic environment. Human listeners appear to develop expectations about room echoes based on their experience of the environment (131), and while they can perceptually separate sound sources from reverberation, this ability is impaired if the environmental statistics deviate too severely from natural values (132). This suggests that exposure and adaptation to long-term reverberation statistics are involved. This plasticity in processing the acoustical properties of the environment may be subserved by adaptation of neuronal receptive fields to changing reverberation statistics, which has been demonstrated in the auditory cortex of ferrets (133, 134).

### 3.5. Functional Advantages of Adaptation to Stimulus Statistics

Adaptation to stimulus statistics across a wide range of timescales, from evolution, to development, to rapid sensory adjustment, is highlighted in theories of sensory coding as the primary means by which the brain is able to adjust its representations to reflect the statistical structure of the outside world. As a result, the brain can process sensory features in ways that are appropriate for performing perceptual tasks and construct neural codes that represent the world effectively and efficiently.

The proposal that adaptation is matched to the properties of sensory stimuli has been of interest since early investigations observed that adaptive mechanisms in the fly visual system had access to multiple timescales and that the timescale of adaptation depends on the design of the experimental paradigm used to probe it (82) (see also Refs. 135, 136 for related work). Importantly, this suggests that traditional “adapt-and-probe” experiments for assessing firing-rate adaptation are not able to fully characterize adaptation to stimulus statistics and that adaptive processes may have multiple underlying neuronal mechanisms with different temporal properties.

In the human cortex, neurons adapt to recent auditory context over a range of timescales: faster responses are sensitive to a longer period of sensory context than slower responses, and the bandwidth of the adaptive processes is dependent on the spectral content of the sound (137). Models of the responses of ferret cortical neurons are improved by incorporating a preprocessing stage that reflects the time constants of adaptation in IC neurons, and these time constants appear to be matched to the frequency dependence of the temporal correlation statistics of natural auditory stimuli (138). Adaptation of inhibitory fields in the ferret auditory cortex to stimuli with varying levels of reverberation similarly reflects the frequency dependence of the statistics of reverberation in different environments (134).

These results tend to confirm that adaptation is matched to the statistics of natural stimuli. However, there are multiple competing (or perhaps complementary) theories of the precise purpose of this matching. Although the statistical distributions of natural sounds have been explored (139–141), a deeper understanding of the relationship between the statistics of natural sounds and corresponding adaptation in the brain will require further characterization of those sound statistics at the temporal scales over which adaptive processes occur.

#### 3.5.1 Perceptual performance.

If adaptation serves to optimize neural processing to the prevailing sensory conditions, we would expect it to have perceptual benefits. In the visual system, perceptual consequences of adaptation are often manifest as aftereffects, where exposure to a particular visual stimulus results in temporary perceptual changes after that stimulus is removed (reviewed in Ref. 49). Aftereffects are not as common or salient in the auditory system, although some examples have been studied (107, 142). Furthermore, as we saw in sect. 3.4, adaptation to preceding sounds can shift induce shifts in auditory localization away from the actual source location.

However, adaptation should also bring perceptual benefits, again illustrated in the context of sound localization by the resulting improvement in spatial discrimination (116–118, 120, 122). Similarly, adaptation to sound contrast (which improves neuronal discrimination between sound levels) results in improved sound-level discrimination in human listeners, the magnitude of which can be accounted for by the gain changes observed at both subcortical and cortical stages of the mouse auditory system (106).

Few studies have investigated the physiological and behavioral consequences of adaptation in the same individuals and the findings to date are inconsistent. One study addressed this by inducing adaptation to a continuous noise background and measuring neural and perceptual responses in macaque monkeys (143). The rate-level functions of neurons in the IC were significantly affected by adaptation; however, neurometric and psychometric thresholds were unaffected, suggesting that adaptation did not have a perceptual effect. On the other hand, adaptation to the contrast of background noise in mice has been reported to influence sound detection behavior and cortical gain in a similar way (144). It seems likely that the consequences of adaptation reflect a compromise between direct effects on perception and effects that are beneficial at the neural coding level, as suggested by experiments examining the cortical representation of sound in the presence of background noise (96) (see subsequent sections).

#### 3.5.2. Efficient coding.

Efficient coding is the general principle that sensory systems in the brain optimize their responses and representations to maximize use of the available neuronal resources, subject to relevant constraints (44, 145). The dynamic range adaptation described above can be considered in these terms: neurons adapt their responses to accurately represent the range of recently heard sounds, subject to the constraints on dynamic range that are imposed by the neuronal hardware. A wide range of other constraints has been considered by various authors, such as number of neurons (146), independence of neural representations (147, 148), metabolic cost (149, 150), and representational stability (151).

A rigorous instantiation of the efficient coding hypothesis is to define efficiency as maximizing the mutual information between a neuron’s responses and the encoded stimulus. An influential study (152) showed that, if neuronal responses have a limited dynamic range and neuronal noise levels are constant, then the mutual information can be maximized by matching the dynamic range of the neuron to the range of stimulus values (histogram equalization) in such a way that each response level is used with equal frequency. The responses of large monopolar cells in the blowfly visual system were found to be matched in this way to the distribution of contrasts found in the visual world (152).

This highlights the important idea that, if they are to be efficient, neuronal representations must be matched to the statistical structure of the stimuli they represent on timescales that are relevant to the variation in those stimuli. On long timescales, this may happen through evolution or developmental processes, so that neuronal responses are matched to the long-term average statistics (153). However, stimulus statistics also vary on short timescales, and matching these statistics requires dynamic adaptive processes that are capable of sampling the recent stimulus statistics and adjusting neuronal responses accordingly. Neuronal adaptation therefore enables sensory information to be represented optimally in the brain despite fluctuations over time in the statistics of the environment.

Early theories largely focused on stimulus representation under various efficiency constraints, by looking at the quality of stimulus reconstruction from neuronal responses. More recent work has tended to investigate the capacity of sensory coding to support tasks that animals need to perform, such as inference (154), prediction (155), or both (156).

#### 3.5.3. Normalization.

The adaptation of gain in ferret cortical neurons to changes in sound contrast is usually compensatory, i.e., decreases in contrast lead to increases in gain (97). This simple, normative relationship suggests that the cortical population may implement a normalized representation of sounds. In a fully normalized representation, neurons would represent the structure of a stimulus in a way that is invariant to changes in the (mean and) variance of the stimulus.

Contrast normalization has been proposed in the visual system, where neurons are similarly subject to contrast-dependent changes in sensitivity (157). Although gain control in the auditory system does not represent complete normalization, i.e., the change in gain does not fully compensate for the change in contrast, this process seems to behave in similar ways in the visual and auditory cortices. In both cases, the gain changes can be described by identical equations (97, 157). The similarities between gain control processes in multiple systems are sufficiently striking that it has been suggested that contrast gain control may be a canonical neural computation (99). In the visual system, contrast is computed locally, so that the gain of each neuron is determined by the contrast of the visual image over an area of the retina close to the neuron’s receptive field (158), rather than over the entire retinal image. Similarly, in the auditory cortex, the gain of each neuron is determined largely by the contrast at sound frequencies that are within the neuron’s receptive field (105), suggesting that contrast is calculated in a comparable fashion in these two sensory modalities.

It has been suggested that contrast normalization offers advantages for population coding. In both the visual and auditory systems, it is thought to be desirable for efficient coding of natural scenes (159). Since the contrast of natural images is correlated across space and time, normalization by stimulus contrast reduces the redundancy of the neural code (160, 161). It may also have a role in the development of distributed representations whereby neurons become tuned to different features of sensory input (162). Furthermore, contrast normalization results in population codes that can be decoded easily using a linear classifier (163) and that can exhibit winner-take-all behavior depending on the relative contrast of different stimuli that are presented simultaneously (164). The latter is a feature that is frequently required by object-recognition algorithms and computation of stimulus saliency (165), as well as by models of attention (166).

Selective attention modulates the representation of stimulus information in early sensory cortex in a manner consistent with normalization processes. Focusing on one speaker in a “cocktail party” situation leads to enhanced tracking of the attended speech stream in neural activity recorded from the human auditory cortex (167). Likewise, in the visual system, it has been proposed that covert spatial attention (the ability to process information preferentially at a particular point on the retina without moving the eyes) may be subserved by a gain control process, which increases the responses of neurons with receptive fields in the attended location, thereby conferring a processing advantage, and decreases responses to unattended locations. It is conceivable that attentional gain control (168) might use the same neuronal circuits as contrast gain control; such an arrangement has been proposed in the visual system (166). In this case, an additional (probably) top-down input to the gain control circuit would be required, modulating the effect of contrast.

#### 3.5.4. Noise tolerance.

One important functional consequence of adaptive processes that implement normalization (even imperfectly) is that they offer another way of enhancing the representation of sounds in background noise. Research investigating the responses of neurons to speech-in-noise stimuli (96) has suggested that the combined effect of mean level and contrast adaptation is to minimize the responses of cortical neurons to a statistically stationary background sound. The remaining neuronal responses mainly depend on the foreground speech sound and are relatively invariant to the contrast of the speech. This enables cortical neurons to represent complex sounds in a fashion that is robust to the presence of background noise and this capacity builds up along the auditory pathway (96). More recent work in guinea pigs has confirmed that cortical neurons are more tolerant to changes in noise level than those found subcortically (169) and has further shown that adaptation to the background noise level improves neurometric discrimination performance, particularly in the MGB and IC.

Experiments have also been carried out to investigate how the cortical representation of speech and vocalizations is affected by the presence of wider ranges of noise, including white and naturalistic (pink) noise and reverberation (133). Various computational models were trained to reconstruct the spectrograms of heard stimuli based only on the neuronal responses recorded in ferret auditory cortex. Importantly, a static decoding model was unable to suppress the noise. Instead, dynamic models were required, suggesting that dynamic adaptive processes (synaptic depression and/or feedback gain normalization) are an essential part of the neuronal mechanisms that achieve noise tolerance.

In humans, related work has combined electrocorticography recordings with behavioral experiments to show similar adaptation to real-world (jet plane, city, and bar) background noise conditions (170). Adaptation suppressed the neuronal representation of noise and enhanced the representation of phonetic differences in the foreground speech sound. The observed adaptation was found not to depend on attentional focus, indicating a potential link to the animal studies, which have often been carried out under anesthesia. Decoding analyses using magnetoencephalography (MEG) in humans have shown that adaptation results in stable encoding of speech against changing backgrounds (171). Furthermore, more recent work using functional MRI suggests that invariance to background noise may be a signature of the more sophisticated auditory process happening at higher levels of the auditory hierarchy (172).

Studies of noise adaptation in animals have also provided insights into the local circuit basis for perceptual changes that have been observed in the presence of background sounds. Thus, noise adaptation in A1 provides a potential neuronal basis for the improvement in tone-in-noise detection thresholds in humans when coherently modulated sidebands are added to the noise masker (173). Like the release from masking found for speech-in-noise recognition noise by human listeners (74, 174), prior adaptation to the noise results in substantial comodulation masking release in mouse A1, which is dependent on cortical activity during the priming period (173). Moreover, the presence of continuous broadband noise has been found to improve tone discrimination for small frequency differences in mice and this behavioral improvement could be replicated by optogenetically activating parvalbumin-positive interneurons to make A1 tuning curves resemble those recorded in the presence of noise (175).

While the capacity of neurons to adapt to the statistics of sounds reaching the ears is critical for hearing in noisy environments, attending to a particular sound source also helps to move other auditory signals that are present at the same time into the perceptual background (46). This has been illustrated by recordings from neurons in the auditory cortex of ferrets trained to discriminate target tones against background noise or to discriminate between different tones or tone complexes (176–178). These studies have shown that the gain and shape of the spectrotemporal receptive fields of cortical neurons can change within a few minutes of beginning the task in ways that appear to enhance the contrast between the two stimulus categories and presumably, therefore, improve perceptual discrimination. Indeed, the magnitude of the spectrotemporal receptive field plasticity has been found to correlate with task performance, implying a direct relationship between levels of attention and the extent of these physiological changes (176).

## 4. STIMULUS-SPECIFIC ADAPTATION

In sect. 2.3, we saw that presentation of one sound can alter the response of auditory nerve fibers to a second sound. The impact of stimulation history on auditory responses has also been explored at the level of the auditory midbrain (179) and cortex (109, 180–182) by presenting pairs of sounds. These studies have revealed stimulus-specific contextual effects, i.e., the way in which the response to the second sound changed was found to be dependent on the physical properties, such as the frequency and level of the preceding stimulus, as well as the interval between the two sounds. Adaptation resulting from the spiking history of the neurons cannot explain the complexity of the observed interactions, indicating that there is more to this than peripheral forward masking.

**FIGURE 6.**
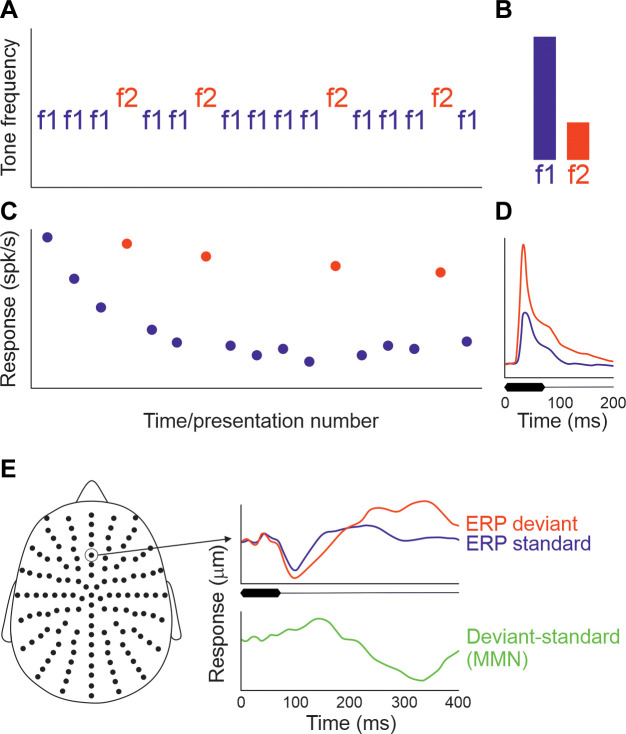
Stimulus-specific adaptation and the mismatch negativity. *A*: in the classical oddball paradigm, a sequence of tones of 2 different frequencies, f1 and f2, is presented, with 1 of the tones (f1 in this case) having a higher presentation probability (the “standard” stimulus), while the other tone (f2, the oddball or “deviant” stimulus) is presented less frequently. *B*: relative probability of the 2 tones in the sequence shown. The order of the stimuli is also reversed, so that f1 becomes the deviant and f2 the standard, to control for the relative sensitivity of neurons to each tone frequency, while other stimulus sequences (not shown) that control for stimulus expectation and rarity allow genuine sensitivity to stimulus deviance to be assessed. *C*: response of a hypothetical auditory neuron to the stimulus sequence shown in *A*. Note that the firing rate to repeated presentations of f1 declines over time (“repetition suppression”), whereas much less adaptation occurs for f2, resulting in a larger average response to the deviant sound than to the standard (*D*). *E*: human event-related potentials (ERPs) measured at the channel indicated in an array of scalp electrodes in response to standard and deviant tones. The mismatch negativity is the difference wave at this location, obtained by subtracting the average response to the standard tones from the average response to the deviant tones. *E* is modified from Ref. 185 (in accordance with http://creativecommons.org/licenses/by/4.0/).

Another well-known example of the context dependence of neuronal responses at higher levels of the auditory pathway is stimulus-specific adaptation (183, 184). This is typically investigated using an oddball paradigm in which a repeating tone of one frequency is occasionally intermixed with a tone of a different frequency. While the responses of neurons to the common or standard tone are reduced over time, the responses to the lower-probability tone decline less or not at all (FIGURE 6, *A*–*D*). In other words, this form of response adaptation does not generalize and is therefore stimulus specific. Strictly speaking, the reduced responses to repeated presentations of the standard stimulus are more akin to habituation (183) and are also often referred to as repetition suppression (186, 187). However, the term stimulus-specific adaptation has been widely adopted and the stimulus specificity of this phenomenon sets it apart from firing-rate adaptation resulting from the intrinsic properties of the neurons or, as we saw earlier, in the auditory nerve, where synaptic depression at the hair cell synapse results in a generalized loss in excitability.

Like other forms of adaptation, stimulus-specific adaptation has been described in both the visual (188–191) and somatosensory (192, 193) pathways but has been a topic of particular interest in the auditory system (183, 184, 194). In part, this reflects the greater temporal precision of auditory processing, particularly in comparison to the visual system (195). However, interest in stimulus-specific adaptation also stems from its similarity to the mismatch negativity; the difference between the human auditory evoked potentials evoked by standard and rare stimuli presented in an oddball sound sequence (185, 196, 197) (FIGURE 6), which is altered in a number of neurodevelopmental and psychiatric disorders (185). Both stimulus-specific adaptation and the mismatch negativity highlight the sensitivity of the auditory system to novel or unexpected sounds occurring amidst a stream of other sounds. Investigating their underlying basis can therefore provide important insights into auditory scene analysis and particularly how different sound sources are segregated.

### 4.1. Stimulus-Specific Adaptation, Deviance Detection, and Predictive Coding

Stimulus-specific adaptation in the auditory system has been extensively studied in A1, where it was first observed (198–208). As with other forms of adaptation, it is present in both awake and anesthetized animals and is most commonly demonstrated by presenting a sequence of standard tones of one frequency in which a rare tone of a different frequency is embedded, with the same stimulus eliciting a larger response when it is rare than when it is frequent (FIGURE 6, *A*–*D*). Various mechanisms have been proposed for stimulus-specific adaptation, with one early prominent idea being that it can be accounted for by a model based on adaptation of narrowly tuned frequency channels (183, 209). The premise of this model is that adaptation occurs when inputs tuned to the standard frequency are repeatedly activated, possibly as a result of synaptic depression, whereas other inputs, including those preferring the rare stimulus frequency, are less adapted. Postsynaptic integration of these differentially adapting inputs should therefore result in stimulus-specific adaptation.

While this model can account for certain properties of stimulus-specific adaptation, a series of studies using different tone sequences as the standard, based on those used as controls in mismatch negativity experiments, has demonstrated that this is not the full explanation for the larger response elicited by the rare or deviant stimulus (205, 208, 210–212). These control stimuli were adopted in an effort to determine the relative contributions of the presentation probability (i.e., the rarity) and predictability of the stimuli on the responses. For example, compared to a sequence of standard tones of the same frequency, the “adaptation load” (183) can be reduced by including the rare frequency within a sequence of tones of many widely spaced frequencies, all with equally low probability. When the tones are presented in a random order, this “many-standards” control also removes any expectations about what the next stimulus might be.

These studies have revealed that responses to deviant tones in the oddball condition tend to be larger than expected on the basis of adaptation of narrowly tuned frequency channels (183, 208, 210–212). Together with evidence from experiments employing oddball sequences that comprised either a fixed or variable number of standards between each deviant (213), they suggest that A1 neurons can encode unexpected sounds that deviate from the statistical regularity of the standard tone sequence, rather than that they are simply sensitive to rare sounds. Although some early studies that also included a “many-standards” control came to a different conclusion (202, 203), it now seems clear that A1 neurons are genuinely sensitive to sound deviance.

Deviance detection is also a defining property of the mismatch negativity (184, 197, 214), and growing evidence from both human and animal studies suggests that this represents a prediction error signal (184, 185, 208, 215). This is based on the influential predictive coding framework, which postulates that predictions of current sensory inputs made by higher level brain regions are compared with the actual signals provided by lower levels in the processing hierarchy (216). Applying this to stimulus-specific adaptation and the mismatch negativity, responses to repetitive, predictable stimuli will be suppressed (as a result of adaptation), whereas mismatches between incoming sensory inputs and a memory trace of previous sounds should produce a prediction error signal in the form of the larger response to a deviant sound (184, 185). This, in turn, is believed to be fed forward to higher levels and used to update the brain’s model for predicting auditory inputs. Furthermore, rather than just being a consequence of neural fatigue resulting from repeated presentations of the same stimulus, it has been proposed that repetition suppression, which can be modulated by contextual factors, such as attention, may play a role in this process by optimizing predictions about the content and precision of sensory inputs (217).

### 4.2. Can We Equate Stimulus-Specific Adaptation with the Mismatch Negativity?

The issue of whether stimulus-specific adaptation in auditory neurons recorded in animals provides a correlate for the scalp-recorded mismatch negativity has attracted considerable attention (184, 218). Both measures are preattentive and likely reflect bottom-up processing of sound deviance to the extent that they are present under general anesthesia, but the mismatch negativity can be elicited by violation of a wider range of stimulus parameters, including frequency, level, duration, and location, and by a silent gap in a regular sequence of sounds (197). It also peaks much later (from 100–250 ms) after deviant onset than the latency at which stimulus-specific adaptation is most commonly recorded in A1. Although a late response component measured in mouse A1 neurons was found to show deviance sensitivity with many of the same properties as the mismatch negativity (205), it is widely accepted that the latter originates in nonprimary auditory areas (184, 196, 218, 219).

**FIGURE 7.**
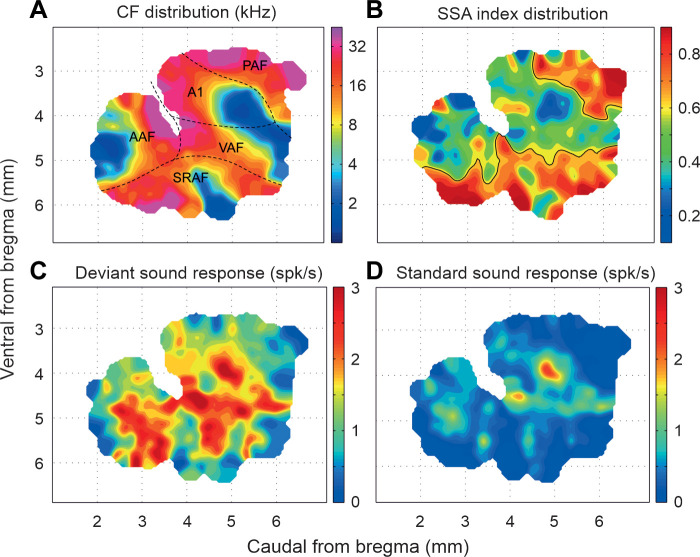
Variation in stimulus-specific adaptation across the rat auditory cortex. *A*: location of five auditory cortical areas (A1, primary auditory cortex; AAF, anterior auditory field; VAF, ventral auditory field; SRAF, suprarhinal auditory field; PAF, posterior auditory field) showing the distribution of characteristic frequencies (the sound frequency to which the recorded neurons were most sensitive). All five areas are tonotopically organized, but A1, AAF, and VAF are considered to be primary or lemniscal areas, whereas PAF and SRAF are secondary or nonlemniscal areas. *B*: distribution of the “common stimulus-specific adaptation (SSA) index,” which compares the magnitude of the neuronal response to standard and deviant tones, across these cortical areas. Stimulus-specific adaptation is strongest in the nonlemniscal cortical fields. *C*: cortical distribution of responses to deviant tones. *D*: cortical distribution of responses to standard tones. Much stronger adaptation has been found in the nonlemniscal cortical fields, where average responses recorded to the standard tone sequence are very small. Modified from Ref. 207 (in accordance with http://creativecommons.org/licenses/by/4.0/).

Stimulus-specific adaptation in A1 neurons and the middle-latency response (10–50 ms) of the auditory evoked potential measured in humans both provide indicators of earlier sound deviance detection, and presumably contribute to the subsequent downstream generation of the mismatch negativity (218). A clearer understanding of the relationship between them has been provided by studies in which stimulus-specific adaptation and deviance sensitivity have been compared between different auditory cortical areas in animals (207, 208, 212, 220). While there are some inconsistencies between the findings from these studies, stronger stimulus-specific adaptation extending into the latency range of the mismatch negativity (207) and larger prediction error signals (208, 220) have been reported in nonprimary fields in rats (FIGURE 7). In particular, a recent study reported that neurons in the posterior auditory field in this species show less adaptation to repeated presentations of a standard tone and stronger prediction error signals than neurons recorded in other auditory cortical areas, including A1 (220). Moreover, mismatch responses recorded in the medial prefrontal cortex of rats are more robust, longer lasting, and more closely resemble predictive error signals than those recorded in the auditory cortex (221). Thus the relative contributions of adaptation and deviance detection to context-dependent processing of sound sequences appear to be organized in a hierarchical fashion both across different auditory cortical areas and beyond.

### 4.3. Stimulus-Specific Adaptation Across the Auditory Pathway

We have so far focused on stimulus-specific adaptation in the auditory cortex. Since adaptation to sound statistics, such as sound level or contrast, arises much earlier in the auditory pathway, it is important to ask whether stimulus-specific adaptation and prediction error representations are also present in subcortical nuclei and how these properties might be generated at the circuit level. Indeed, a neural correlate of deviance detection has been recorded noninvasively from the auditory brainstem in humans (222). This is particularly relevant for stimulus-specific adaptation and deviance detection given the hierarchical nature of predictive coding models, involving an interplay between feedforward and top-down signaling (185, 216).

**FIGURE 8.**
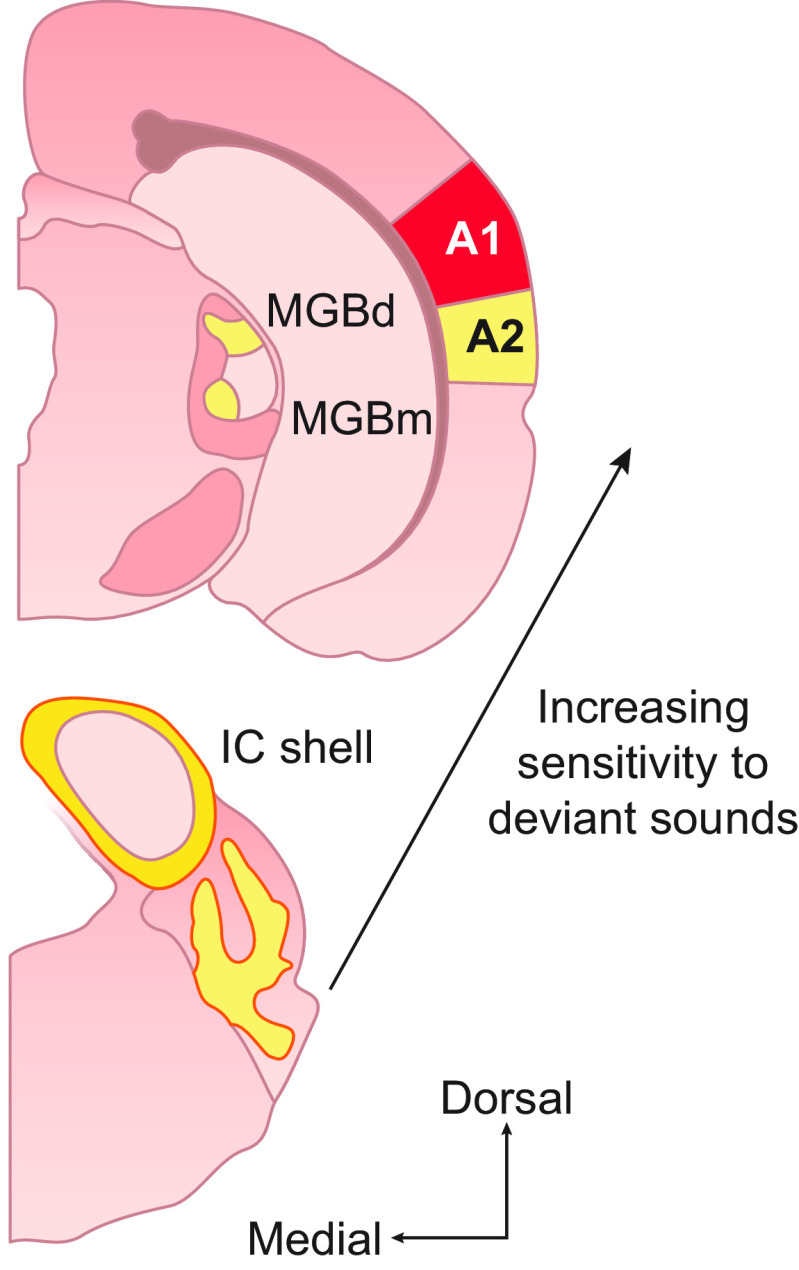
Distribution of stimulus-specific adaptation and deviance detection in the auditory system. Two coronal sections of the mouse brain are represented, with the lemniscal and nonlemniscal auditory regions where these context-dependent properties are found colored in red and yellow, respectively. Stimulus-specific adaptation appears to be an emergent feature of the responses of neurons in the primary auditory cortex (A1), since it is found to a much lesser degree in subcortical structures in the lemniscal auditory pathway that provide the majority of the ascending input to A1. However, stronger stimulus-specific adaptation is found in nonlemniscal regions of the auditory midbrain [the shell of the inferior colliculus (IC)] and thalamus [the dorsal and medial divisions of the medial geniculate body (MGBd and MGBm)], as well as nonlemniscal cortical areas [such as the secondary auditory cortex (A2)]. Stimulus-specific adaptation and deviance detection, which is thought to provide an index of prediction error signaling, increase in a hierarchical fashion along the auditory pathway from the IC to higher level auditory cortex.

While stimulus-specific adaptation is a prominent feature of neurons in A1, attempts to show whether it is also present in the MGBv, which provides their principal source of thalamic input, have produced mixed results (198, 208, 223–227). Stimulus-specific adaptation for tones of different frequencies is found subcortically, but primarily in nonlemniscal regions of the MGB (208, 223, 225) and IC (194, 208, 228–231), and is absent in the cochlear nucleus (232) (FIGURE 8).

Compared to the MGBv and CNIC, the lemniscal areas that we focused on when considering adaptation to stimulus statistics, neurons in nonlemniscal regions of the auditory thalamus (233–236) and midbrain (235, 237–240) are generally characterized by broader frequency tuning, less well-defined or nonexistent tonotopic organization, and a greater prevalence of inputs from other sensory modalities. Moreover, nonlemniscal regions of the MGB, particularly its dorsal division, mainly target nonprimary or “belt” areas of the auditory cortex, rather than A1, while the medial division projects to most auditory cortical areas, terminating principally in cortical layer 1 (241–245). Given that a very small proportion of the ascending auditory input to A1 originates from nonlemniscal thalamic areas, it is likely that stimulus-specific adaptation in A1 arises as a result of local processing, rather than being inherited from the thalamus. This is supported by the finding that stimulus-specific adaptation is stronger, as measured by the contrast between neuronal responses to the oddball and standard tones, in the superficial and deeper layers of A1 than in its principal thalamorecipient layers (200).

Neural network modeling has shown that recurrent intracortical synaptic depression, rather than synaptic depression of feedforward thalamocortical inputs, can account for many of the properties of stimulus-specific adaptation, including deviance sensitivity, that have been observed in recordings from A1 (246). It is also thought that local neuronal inhibition may contribute, since stimulus-specific adaptation in the auditory cortex is exhibited by both excitatory pyramidal neurons and by a range of inhibitory interneurons (205, 206, 247). On the basis of the changes observed following optogenetic suppression of these interneurons, it seems clear that they are not responsible for generating stimulus-specific adaptation in pyramidal neurons. Intracortical inhibition can regulate the strength of stimulus-specific adaptation, however, by differentially modulating A1 responses to deviant and standard sounds (206, 247). While further research is needed to understand how different types of inhibitory interneuron interact to shape the sensitivity of the cortex to unexpected sounds, other mechanisms, such as synaptic depression or firing-rate adaptation due to activation of intrinsic membrane currents (248), are also involved in determining the response dependence of cortical neurons on their previous history of stimulation.

Regardless of its cellular basis in A1, the finding that stimulus-specific adaptation is relatively weak in the MGBv contrasts with other forms of adaptive coding, such as contrast gain control, which is displayed by neurons in lemniscal regions of the auditory midbrain, thalamus and cortex (96, 106). This suggests that different forms of adaptation may arise at different processing levels in the auditory system. Nevertheless, conclusions about their respective origins should be tempered since adaptation to sound contrast and other statistical properties has yet to be explored in any detail in nonlemniscal regions of the IC and MGB. Where measurements have been made at several processing levels along the auditory pathway, however, it is clear that each type of adaptive coding is hierarchically organized. Thus, as we saw in sect. 3.5.4, changes in neuronal adaptation to mean level and contrast between the CNIC and A1 in ferrets appear to result in representations of complex sounds that become increasingly tolerant to the presence of background noise (96). Similarly, stimulus-specific adaptation and prediction error signals progressively increase in magnitude between the nonlemniscal regions of the IC, MGB, and auditory cortex (208). In fact, if spectrally balanced tone clouds are used as stimuli to rule out any contribution of frequency-specific adaptation, robust stimulus-specific adaptation is observed in early responses recorded in A1 but not at any subcortical level (227).

While these findings point to a key role for the auditory cortex in adaptive coding, the existence of stimulus-specific adaptation for tones of different frequencies in the nonlemniscal shell of the IC shows that sensitivity to an unexpected change in frequency is established at a lower level of the pathway. Local GABAergic inhibition has again been implicated in regulating the gain of stimulus-specific adaptation in both the IC (249) and MGB (250) but cannot account for the generation of these responses. Nevertheless, inhibitory inputs from the thalamic reticular nucleus appear to contribute to stimulus-specific adaptation in the MGB (224), so further research is needed to determine whether stimulus-specific adaptation and deviance detection are computed as a result of local processing in these brain areas.

#### 4.3.1. Descending signals and stimulus-specific adaptation.

Because nonlemniscal regions of the auditory midbrain and thalamus are heavily innervated by descending projections from the auditory cortex (251, 252), we also have to consider whether stimulus-specific adaptation and deviance detection are controlled by the corticofugal system. A range of effects on subcortical repetition suppression and stimulus-specific adaptation has been reported following deactivation of the auditory cortex, the magnitude of which depends to some extent on the target structure and the methods used to manipulate the descending pathways (226, 253–255). While this makes it challenging to draw firm conclusions, it is interesting to note that IC neurons that exhibit stimulus-specific adaptation are more likely to receive cortical inputs than those that do not (256). Furthermore, while cooling the cortex did not affect the magnitude or time course of stimulus-specific adaptation in the majority of neurons recorded in the MGB (254) or IC (253), the gain of the neurons changed according to how much stimulus-specific adaptation they exhibited.

Additional evidence for a role for descending inputs in modulating the sensitivity of stimulus-specific adaptation has recently been provided by a reduction in prediction error signaling in the IC that results from more targeted optogenetic suppression of corticocollicular neurons (255). There is therefore growing evidence for the possibility that an interplay between cortical and subcortical circuits provides a substrate for comparing descending predictions of auditory input, in this case a sequence of repeating tones, with ascending sensory signals, generating a prediction error when deviant sounds are encountered.

## 5. ADAPTIVE CODING ACROSS THE LIFE SPAN

The various forms of adaptation that we have considered in this article have all been investigated primarily in adulthood and relatively few studies have looked at their development or whether they change with age. In this section, we consider what is currently known about the developmental emergence of adaptive coding properties in the auditory system and the way those properties are shaped by experience and learning, before looking at the effects of hearing loss on neuronal adaptation in sect. 6.

Evidence that the auditory system is optimized to process natural stimulus statistics is provided by perceptual studies in humans. For example, listeners tend to group together naturally co-occurring sound features, which are more likely to be heard as a single source than pairs of features that do not commonly co-occur (153). Furthermore, the ability of listeners to perceptually separate sound sources from the accompanying reverberation is impaired if the environmental statistics deviate markedly from the local values experienced in a range of inner-city and rural locations (132), suggesting that that prior experience of these statistics is important for perception. This dependence could arise over the course of development, and perhaps also into later life, or as a result of longer term shaping of the brain by evolution. Distinguishing between these possibilities is challenging, but recordings in ferrets suggest that individual experience is likely to be important since the correlation structure of spontaneous activity in the visual cortex adapts during development to covertly represent priors about the statistical structure of the natural visual environment (257).

### 5.1. Shaping Neuronal Adaptation by Experience

Among the various response properties that undergo maturational changes, the capacity of mammalian auditory neurons to follow periodic trains of clicks has been shown to improve over the course of postnatal development, indicating that adaptation to repetitive sounds becomes less pronounced with age (258, 259). Furthermore, the following response appears to mature at progressively later ages from the auditory nerve to the auditory cortex. In a similar vein, neurons in the NCM (caudo-medial nidopallium), a region of higher level auditory cortex in songbirds, show a lower rate of adaptation in adults than in juveniles to presentations of either the song they have been tutored with or novel conspecific songs (260). The more persistent response of these neurons in adult zebra finches may reflect their greater familiarity with the song stimuli, particularly during song learning. Indeed, NCM neurons in adult zebra finches show a more gradual reduction in response with repetition of familiar than novel songs (261, 262), highlighting the stimulus-specific nature of this adaptation.

Intriguingly, a difference in the rate of neuronal adaptation for tutor song compared with novel conspecific songs was only found in adult birds that had been experimentally tutored as juveniles and this correlated with their capacity to imitate the tutor song (262). These findings therefore suggest that the extent to which NCM neurons adapt to repetitive song is indicative of the birds’ auditory memory for the tutor song and that adaptation is shaped by auditory experience. In fact, this plasticity seems to continue into adulthood, since the rate of neuronal adaptation in the auditory forebrain was found to covary with the speed with which zebra finches learn on an operant training task to discriminate different song stimuli (263).

While these studies have examined the development and plasticity of neuronal adaptation to repeated presentations of the same stimulus, i.e., repetition suppression, they have not addressed stimulus-specific adaptation per se by using oddball paradigms to compare responses to standard and deviant stimuli. In humans, the mismatch negativity can be recorded in newborn infants (264, 265) and even from the fetal brain (266). Although broadly similar to the response recorded in adults, differences in the temporal properties and scalp distribution of the mismatch negativity are present at these earlier ages. As far as we are aware, comparable developmental studies on neuronal stimulus-specific adaptation and deviance detection have not been carried out in animals. However, the prevalence and strength of stimulus-specific adaptation in A1 was found to be reduced by exposing young rats to continuous broadband noise for several weeks, a procedure that disrupts cortical development, whereas no differences were observed between control animals and an adult noise-exposure group (267).

There is some evidence that early auditory experience shapes signal-in-noise processing in the mammalian brain. Rearing rats in the presence of spectrotemporally modulated noise resulted in improved detection of vocalizations in noise, as assessed both behaviorally and in a neuronal decoding analysis (268). This work suggests that rearing in noise reduced neuronal responses to sounds with the statistics of the noise and further showed how these changes can be described in terms of changes in the multidimensional receptive fields of neurons in auditory cortex (269).

Research in humans also indicates that sensitivity to statistical regularities in sounds is shaped by experience, potentially giving rise to individual differences in adaptive coding. In these studies, auditory brainstem response measurements were made to a speech syllable that was either presented repeatedly (and therefore predictably) or in a pseudorandom order along with other speech sounds. The sensitivity of brainstem responses in children to the ongoing context in which the stimulus was presented was positively correlated with behavioral measures of speech-in-noise perception (270) and with their musical and literacy skills (271). This research also demonstrated that the sensitivity of subcortical circuits to statistical regularities can be changed in different ways by musical experience (272) or by auditory training (273). Furthermore, in trained musicians, the extent of the observed subcortical enhancement in the detection of predictably occurring speech correlated with an improvement in their ability to hear speech in background noise (272). Thus, in addition to demonstrating that learning can alter how the auditory system responds to stimulus context, these studies provide further evidence for a link between adaptation to stimulus statistics and signal-in-noise perception.

### 5.2. Neurodevelopmental Disorders

An important and underexplored question concerns the possible relationship between abnormalities in auditory adaptation and neurodevelopmental disorders, such as dyslexia or autism. There is growing evidence that individuals with dyslexia show reduced repetition-induced adaptation in auditory and other cortical areas (274, 275), which is apparent for both linguistic and nonlinguistic sounds, as well as reductions in both neural prediction error signals (276) and perceptual adaptation to speech (277). Furthermore, better reading skills in adults and children with dyslexia have been found to be associated with greater repetition-induced neural adaptation (274). Behavioral and electrophysiological evidence suggests that impaired adaptation in the auditory cortex is also linked to a poorer ability to learn sound regularities, which, in turn, may result in deficits in sound category formation and phonological processing in dyslexia (278).

While these studies have emphasized changes in cortical processing, differences between individuals with dyslexia and typically developing participants have also been noted at the level of the thalamus (278). Additional evidence for subcortical changes is provided by the finding that context-dependent brainstem encoding of speech is impaired in children with developmental dyslexia (270), which may represent another example of the importance of descending corticofugal influences on sound processing at lower levels of the auditory pathway.

### 5.3. Aging and Neuronal Adaptation

An age-dependent decline in sound processing takes place throughout the auditory pathway (279–281). While some of these changes are the direct consequence of peripheral hearing loss (see sect. 6), it is clear that aging-related changes also take place in the response properties of auditory neurons in the brain, which are often associated with loss of synaptic inhibition. Many people find that listening in noisy backgrounds becomes more difficult as they get older (282), and deficits in sound encoding in noise have been observed with aging at both subcortical (283) and cortical processing levels (284). Although various factors, such as impaired temporal processing, are likely to contribute to this age-related decline, it is also possible that changes in the capacity of the brain to adapt to stimulus statistics are involved.

In keeping with the animal studies described in sect. 3.1, MEG has been used to demonstrate that the human auditory cortex adapts to sound-level statistics (285). However, adaptation is less complete in older adults than in younger adults, suggesting that, with increasing age, background sounds are likely to interfere more with auditory processing. Similarly, recordings of the scalp-evoked middle-latency response (286) and the later mismatch negativity (287–289) suggest that older adults are less sensitive to sound deviance. An age-related decrease in the strength of stimulus-specific adaptation has also been reported in rat A1, due to much less suppression in the response to repeated standard tones than in younger animals (290). Similar effects of aging on the temporal dynamics of adaptation have been found in the human auditory cortex too (291, 292), with less reduction in the amplitude of event-related potentials in response to repeated sounds in older adults than in younger adults (293, 294).

While these findings indicate that the sensitivity of the auditory cortex to the regularity of sound sequences is impaired in older individuals, this may not be a permanent change, since responses of older rat A1 neurons to oddball tones can be enhanced by auditory training (290). Moreover, it is possible that the effects of aging on stimulus-specific adaptation are limited to the cortex, since no differences have been found in the MGB between younger and older animals (295).

## 6. ADAPTIVE CODING FOLLOWING HEARING LOSS

Given the very high incidence of hearing disorders, with the World Health Organization estimating that by 2050, as many as 2.5 billion people, or 1 in 4 individuals, will have some form of hearing loss (World Report on Hearing; Geneva: World Health Organization, 2021; License: CC-BY-NC-SA 3.0 IGO), it is important to also consider the impact of a reduction in peripheral input on adaptive coding in the brain. In particular, the likelihood of experiencing hearing loss increases substantially in older people, adding to the direct effects of aging on central auditory processing and posing a particular challenge for listening in noisy backgrounds.

There are two ways of looking at the interplay between hearing loss and adaptation in the brain. First, if the adaptive capabilities of the brain have evolved to cope with the range of environmental statistics that are typically encountered, can they also accommodate changes in input associated with sensory impairments? This perspective has gained traction in studies of vision, where adaptation to optical aberrations or to wearing glasses may help to normalize visual coding and perception across individuals (49). However, as we outlined at the start of this article, longer term, experience-dependent plasticity is known to play a critical role, particularly during development, in adjusting neural circuits to growth-dependent changes in sensory inputs and is also triggered by hearing loss (40, 43, 296). Consequentially, as we saw with the effects of musical and other forms of experience during development on context-dependent processing (sect. 5.1), plasticity triggered by hearing loss is also likely to alter the capacity of the brain to adapt to sound statistics.

This is illustrated, for example, by the adaptive plasticity in spatial hearing induced by raising animals with a conductive hearing loss in one ear. While binaural cues normally dominate sound localization in the horizontal plane, ferrets raised with one ear occluded recover their ability to localize sound primarily by becoming more dependent on the monaural spatial cues provided by the normally hearing ear and this is paralleled by equivalent plasticity in A1 (16). However, this study also demonstrated that the reweighting of different spatial cues induced by monaural deprivation disappears as soon as normal hearing is experienced, illustrating the context-specific nature of the plasticity, which therefore helps to maintain accurate sound localization under both normal and abnormal hearing conditions.

Damage to the auditory periphery, such as loss of cochlear hair cells or of the spiral ganglion neurons that supply their afferent innervation, has been shown to result in a host of changes in auditory brain regions (43, 296). This plasticity includes increased neural activity at higher levels of the auditory pathway, which has been linked to both hyperacusis, an abnormally high sensitivity to sound, and to tinnitus, where phantom sounds are perceived (297, 298). These gain changes are thought to compensate for the reduction in afferent input resulting from peripheral damage and have been observed in the IC (297, 299), the MGB (300), and particularly the auditory cortex (297, 299, 301–303), including in its descending projection to the midbrain (304).

Hearing loss can also alter the adaptive properties of auditory neurons. Auditory nerve fibers show faster firing-rate adaptation during tone presentation and a slower recovery from adaptation following noise-induced sensorineural hearing loss (305), changes that are presumably propagated to auditory brain areas. Moreover, the capacity of auditory cortical neurons to follow repeated stimulation is changed, with less adaptation observed across trains of injected current pulses in neurons recorded in vitro from gerbils that had experienced either a sensorineural or conductive hearing loss in infancy (306).

**FIGURE 9.**
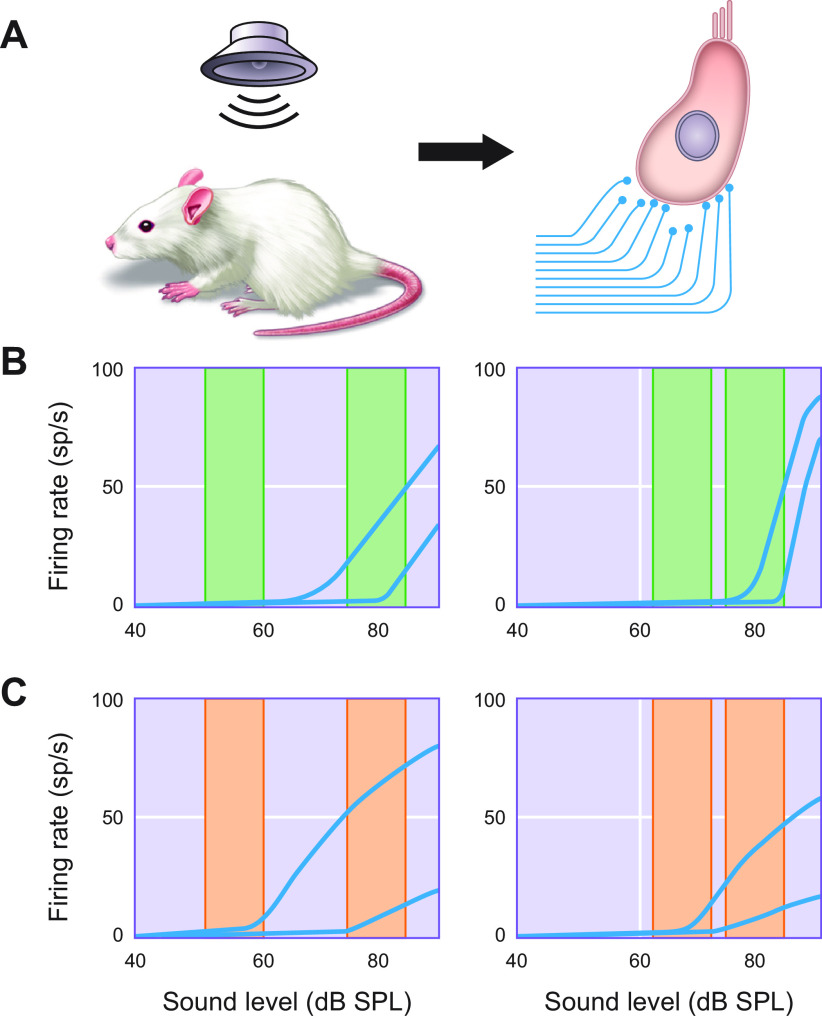
Cochlear synaptopathy impairs neural adaptation to mean sound level. *A*: exposing mice to octave-band noise (8–16 kHz) at 100 dB SPL for 2 hours from an overhead loudspeaker is thought to result in a loss of a proportion of the synapses between cochlear hair cells and auditory nerve fibers (assessed from changes in the gain of the auditory brain response). *B* and *C*: the blue lines represent example rate-level functions recorded in the inferior colliculus (IC) following adaptation to the mean level of broadband noise stimuli in which 80% of the levels presented were drawn from a narrow high-probability region (vertical bars) that switched between 2 values centered at 56 and 80 dB SPL (*left*) or at 68 and 80 dB SPL (*right*). Data from a control mouse are shown in *B* and from a noise-exposed animal in *C* (see FIGURE 2 for further examples of how IC neurons normally adapt to mean sound level). In both groups of mice, IC responses adapted to the current acoustic environment by shifting the threshold of their rate-level functions towards the corresponding high-probability region. However, adapted thresholds in noise-exposed animals were generally lower than in control mice, particularly for the relatively loud sound levels centered at 80 dB SPL. Modified from Ref. 307 (in accordance with http://creativecommons.org/licenses/by/4.0/).

Adaptation to sound-level statistics is also vulnerable to hearing loss. This has been demonstrated in a study in which mice were exposed to noise with the aim of inducing cochlear synaptopathy, a reduction in the synaptic communication between cochlear hair cells and auditory nerve fibers (307) (FIGURE 9). Despite the loss of the synapses, the hair cells remain intact and so hearing thresholds are largely unaffected, giving rise to the term “hidden hearing loss” (308, 309). Cochlear synaptopathy is known to take place during aging, preceding hair cell loss and threshold increases (310, 311). Following exposure to noise designed to selectively damage high-threshold auditory nerve fibers, IC neurons displayed a reduced capacity to adapt to, and their responses were less informative about, sound-level distributions centered on high mean values (i.e., loud environments) (307). By contrast, adaptation to quieter sound levels remained intact. The neurons also exhibited impaired meta-adaptation on a longer timescale to the overall statistics of the fluctuating acoustic environments. It is possible that a specific deficit in adaptive coding at high sound levels may contribute to the listening difficulties in noisy conditions that are often experienced by people with otherwise normal hearing.

An impairment in the sensitivity of auditory cortical neurons to sounds presented against a background of noise has been demonstrated more directly in a mouse model of cochlear neural degeneration in which the spiral ganglion neurons are largely lost (302). Hyperexcitability of the cortex resulting from a recovery in the responses of excitatory pyramidal neurons and a persistent suppression of parvalbumin-expressing inhibitory neurons helped to compensate for the hearing loss and maintain tone-evoked cortical responses and detection behavior in quiet conditions. However, these changes appeared to disrupt the processes that enable cortical neurons to adapt to background noise. While further research is required to investigate the cellular and circuit basis of adaptive coding, these studies suggest that the processes involved at different levels of the auditory pathway may be particularly susceptible to hearing loss.

## 7. CONCLUSIONS

**FIGURE 10.**
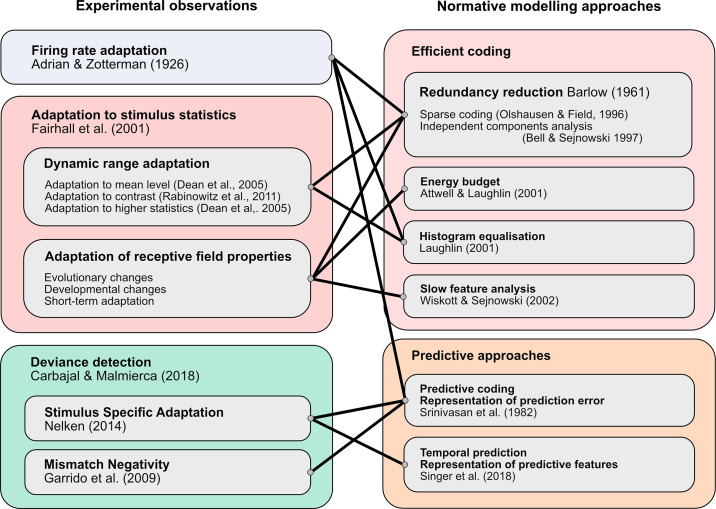
Classification of experimental observations of adaptation (*left*) and associated normative principles (*right*). Examples of studies that exemplify each approach are given. Black lines link each experimental observation with normative principles that have been proposed to account for that observation; this is not exhaustive. Reference numbers are as follows: Adrian and Zotterman (48), Fairhall et al. (82), Dean et al. (88), Rabinowitz et al. (97), Carbajal and Malmierca (184), Nelken (183), Garrido et al (185), Barlow (44), Olshausen and Field (147), Bell and Sejnowski (148), Attwell and Laughlin (149), Laughlin (152), Wiskott and Sejnowski (312), Srinivasan et al. (313), and Singer et al. (155).

The study of adaptation has provided key insights into fundamental mechanisms that underlie the processing of sensory information in the brain. As in other sensory systems, various forms of adaptation are found throughout the auditory system, demonstrating the highly dynamic way in which sounds are processed (FIGURE 10). By continually adjusting neuronal response properties to the current sound statistics, including mean level and contrast, the brain is able to maintain a contextually appropriate and computationally efficient representation of the auditory world. This flexibility contributes to the capacity of auditory neurons to encode a very wide range of sound levels that would otherwise exceed the dynamic range over which their firing rate varies. Furthermore, the benefits of adaptation to sound statistics are particularly apparent when the auditory system is faced with the challenge of segregating target sounds from background noise.

In addition to optimizing the representation of sound as the statistics of sound stimulation vary, adaptation reduces responses to sounds that remain unchanged over time and that are likely to be less informative about the current soundscape in favor of unexpected and potentially more salient sounds. This highlights another important coding principle, namely that auditory neurons show sensitivity to the predictability of sounds in a sequence, which is widely interpreted as support for the theory of predictive coding.

In searching for where adaptive coding takes place in the brain, valuable insights have been obtained into how sound processing is transformed along the auditory pathway and the interplay of ascending and descending signaling. Although some differences exist between species, studies of contrast adaptation and stimulus-specific adaptation are both consistent with the hierarchical emergence of context-dependent coding. However, while contrast adaptation is found in lemniscal auditory brain regions, stimulus-specific adaptation is more prominent in nonlemniscal subcortical areas, with both forms of adaptation being strongly expressed by A1 neurons. In turn, the most noise-tolerant responses and largest prediction error signals are found in the auditory cortex. Knowledge of how adaptation builds up across different stages of processing is critical for constraining the local and network mechanisms involved and paves the way for more detailed investigation of the cellular circuit and biophysical bases for these properties.

While it is clear that adaptive changes in neuronal response properties take place over multiple timescales, which differ across the processing hierarchy, there is growing evidence that the dynamics of adaptation are closely related to the statistical structure of the natural auditory world. A more complete description of natural sound statistics should therefore inform our understanding of which sound properties neurons adapt to and over what time frame. This raises important questions over whether the time constants of neuronal adaptation have arisen over the course of evolution or are shaped by the acoustic experience of individual listeners. Although the relationship between adaptation to sound statistics and learning has so far received relatively little attention, studies in humans indicate that adaptive coding in the auditory brainstem can be influenced by musical experience and by training (most likely as a result of top-down corticofugal modulation), and that these interactions have an impact on speech-in-noise perception. It is also becoming clear that the adaptive properties of auditory neurons are altered in certain neurodevelopmental disorders, such as dyslexia, and change as a result of aging and hearing loss. As our understanding of the factors affecting adaptation grows, we are learning more about the basis for speech perception difficulties in challenging listening environments.

The widespread effects of adaptation on neural coding raise a key question: what is the computational goal of adaptation? Numerous attempts have been made to answer this question through normative explanations of brain function, i.e., hypotheses about the computational principles underlying how a neural system should behave, such as efficient or predictive coding. Such principles are usually implemented in neural network models, and the behavior of computational units in the models are compared with those of real neurons. In many cases, compelling similarities have been demonstrated, which suggest that the principles underlying adaptation in the brain resemble those underlying the models. At present, however, it is unclear which of these candidate theories, which can account for different aspects of adaptation in a variety of systems, is most likely to reflect the way in which adaptive processing in the brain actually works.

Further work is required to develop a deeper understanding of the relationship between different normative accounts, which includes determining which aspects of the models are crucial for making accurate predictions about specific aspects of neural processing and critically evaluating the models by testing their predictions against neural data obtained in different species and from different levels of the sensory pathways. This will also bring us closer to understanding whether adaptation throughout the brain can be understood in terms of a single, unified theory or whether evolution has developed ad hoc solutions for different situations. We believe that combining experimental and computational techniques to evaluate the capacity of different theories to explain the neuronal data offers the best hope for understanding the principles, goals, and consequences of adaptive coding in the brain.

## GRANTS

Our research is supported by a Wellcome Principal Research Fellowship (WT108369/Z/2015/Z to A.J.K.).

## DISCLOSURES

No conflicts of interest, financial or otherwise, are declared by the authors.

## AUTHOR CONTRIBUTIONS

B.D.B.W. and A.J.K. prepared figures; B.D.B.W. and A.J.K. drafted manuscript; B.D.B.W. and A.J.K. edited and revised manuscript; B.D.B.W. and A.J.K. approved final version of manuscript.

## Footnotes

1 It is important to note that we intend the term “optimized” to have a nontechnical meaning, i.e. “made as good as possible.” In other contexts, optimized can imply perfect refinement of a well-defined cost function, subject to known constraints. However, in the context of neuronal representations, there are many competing factors that limit the suitability of any particular representation for any particular purpose. Some of these factors are known and have clear implications (such as biophysical limitations on the firing rate of neurons). Others may be partly understood (such as energetic constraints on the size of neuronal populations). Other factors may be entirely unknown. It is therefore not possible to know whether a particular neuronal representation is globally optimal given all relevant constraints. Furthermore, we know that neuronal representations have not been shaped by a well-defined optimization algorithm; instead, they have been refined indirectly through processes of evolution and development. We therefore intend optimized only to mean that a representation has been made measurably better, to an extent that is likely limited by the vagaries of evolution and development.
